# Brain vitamin D_3_-auto/paracrine system in relation to structural, neurophysiological, and behavioral disturbances associated with glucocorticoid-induced neurotoxicity

**DOI:** 10.3389/fncel.2023.1133400

**Published:** 2023-03-20

**Authors:** Olha Lisakovska, Dmytro Labudzynskyi, Anna Khomenko, Dmytro Isaev, Alina Savotchenko, Ludmila Kasatkina, Serhii Savosko, Mykola Veliky, Ihor Shymanskyi

**Affiliations:** ^1^Department of Biochemistry of Vitamins and Coenzymes, Palladin Institute of Biochemistry, Kyiv, Ukraine; ^2^Department of Cellular Membranology, Bogomoletz Institute of Physiology, Kyiv, Ukraine; ^3^Research Laboratory for Young Scientists, Palladin Institute of Biochemistry, Kyiv, Ukraine; ^4^Department of Histology and Embryology, Bogomolets National Medical University, Kyiv, Ukraine

**Keywords:** glucocorticoids, glucocorticoid-induced neurotoxicity, prednisolone, vitamin D_3_, vitamin D-auto/paracrine system, oxidative-nitrosative stress, behavioral impairments

## Abstract

**Introduction:**

Vitamin D_3_ (VD_3_) is a potent para/autocrine regulator and neurosteroid that can strongly influence nerve cell function and counteract the negative effects of glucocorticoid (GC) therapy. The aim of the study was to reveal the relationship between VD_3_ status and behavioral, structural-functional and molecular changes associated with GC-induced neurotoxicity.

**Methods:**

Female Wistar rats received synthetic GC prednisolone (5 mg/kg b.w.) with or without VD_3_ (1000 IU/kg b.w.) for 30 days. Behavioral, histological, physiological, biochemical, molecular biological (RT-PCR, Western blotting) methods, and ELISA were used.

**Results and discussion:**

There was no difference in open field test (OFT), while forced swim test (FST) showed an increase in immobility time and a decrease in active behavior in prednisolone-treated rats, indicative of depressive changes. GC increased the perikaryon area, enlarged the size of the nuclei, and caused a slight reduction of cell density in CA1-CA3 hippocampal sections. We established a GC-induced decrease in the long-term potentiation (LTP) in CA1-CA3 hippocampal synapses, the amplitude of high K^+^-stimulated exocytosis, and the rate of Ca^2+^-dependent fusion of synaptic vesicles with synaptic plasma membranes. These changes were accompanied by an increase in nitration and poly(ADP)-ribosylation of cerebral proteins, suggesting the development of oxidative-nitrosative stress. Prednisolone upregulated the expression and phosphorylation of NF-κB p65 subunit at Ser311, whereas downregulating IκB. GC loading depleted the circulating pool of 25OHD_3_ in serum and CSF, elevated VDR mRNA and protein levels but had an inhibitory effect on CYP24A1 and VDBP expression. Vitamin D_3_ supplementation had an antidepressant-like effect, decreasing the immobility time and stimulating active behavior. VD_3_ caused a decrease in the size of the perikaryon and nucleus in CA1 hippocampal area. We found a recovery in depolarization-induced fusion of synaptic vesicles and long-term synaptic plasticity after VD_3_ treatment. VD_3_ diminished the intensity of oxidative-nitrosative stress, and suppressed the NF-κB activation. Its ameliorative effect on GC-induced neuroanatomical and behavioral abnormalities was accompanied by the 25OHD3 repletion and partial restoration of the VD_3_-auto/paracrine system.

**Conclusion:**

GC-induced neurotoxicity and behavioral disturbances are associated with increased oxidative-nitrosative stress and impairments of VD_3_ metabolism. Thus, VD_3_ can be effective in preventing structural and functional abnormalities in the brain and behavior changes caused by long-term GC administration.

## 1. Introduction

Naturally occurring glucocorticoids (GCs) and their synthetic analogs, such as prednisolone and dexamethasone, are known to be widely used in medical practice due to their anti-inflammatory, desensitizing, immunosuppressive, and antiallergic properties (Cooper, 2012). However, long-term GC supplementation for the treatment of arthritis, asthma, chronic obstructive pulmonary disease and, especially now, COVID-19 increases the risk of developing a number of adverse complications, including their significant toxic effects on the central nervous system (CNS) (Alan and Alan, 2017). GC-induced neurotoxicity manifests itself in the form of impaired motor and sensory functions, emotional status, as well as integrative functions of the brain, such as memory and learning. These effects are predominantly, but not exclusively, mediated by dysfunction of the hippocampus as this structure is highly sensitive to glucocorticoids (Conrad, 2008). For example, elevated levels of GCs have been shown to cause atrophy of dendritic processes in the CA3 region, loss of hippocampal neurons, and increased neuronal damage in pathological conditions such as seizures and stroke (Patil et al., 2007; Tata and Anderson, 2010). In addition, possible mechanisms of the neurotoxic effect of GC include disruption of GR-associated signaling pathways, cross-links between transcription factors, glucocorticoid receptor (GR) polymorphisms, effects on DNA methylation and other molecular networks involved in switching between autophagy/apoptosis, mitophagy, proliferation/cell differentiation. The target effect of glucocorticoids also covers the functioning of the blood–brain barrier (BBB), astrocytes, microglia, neurons and their dendritic architecture (Obradovic et al., 2006; Fietta et al., 2009; Kim et al., 2010; Kadmiel and Cidlowski, 2013; Sorrells et al., 2014). It is known that GCs can block transendothelial transport of glucose in the CNS, as well as its uptake by hippocampal neurons and glial cells (Horner et al., 1990), thereby disrupting energy metabolism in the brain and affecting the basic functions of neurons. In addition, the negative effects of GCs may be associated with their ability to potentiate the excitotoxic effects of glutamate and reduce the inhibitory effect of GABA in the CNS (Di et al., 2009). However, there have been conflicting data from animal models showing that the glucocorticoid dexamethasone has both neuroprotective and neurotoxic effects in a dose- and time-dependent manner (Abrahám et al., 2001; Lear et al., 2014). These conflicting data raise the question of the known degree of caution in the therapeutic use of glucocorticoids.

Currently, there is no complete clarity regarding the nature of GC-associated changes in the CNS, and the molecular mechanisms underlying the development of GC-induced structural and functional abnormalities in the CNS, cognitive and behavioral disorders are poorly understood. In this regard, difficulties may arise in solving the urgent biomedical problem of finding the most effective pharmacological approaches for the correction and/or prevention of GC-induced neurotoxicity.

Vitamin D_3_ (VD_3_, cholecalciferol) has previously been shown to play an important role as a para- and autocrine neurosteroid that modulates multiple CNS functions, including brain development, neurotransmission, neuroprotection, and immunomodulation (Kalueff and Tuohimaa, 2007). VD_3_ can easily cross the BBB (Won et al., 2015) and realize its biological action in the brain through its own vitamin D_3_-auto/paracrine system (Cui et al., 2017), which consists of several major components. Vitamin D_3_ receptor (VDR) is a key player that binds the hormonally active form of VD_3_, 1,25-dihydroxyvitamin D_3_ (1,25(OH)_2_D_3_, calcitriol). The VDR is a member of the nuclear receptor superfamily and is present in both the developing and adult brains. In the brain of adult rats, VDR is found in neurons, astrocytes (Brown et al., 2003), and oligodendrocytes (Baas et al., 2000) of various brain areas. 25-hydroxyvitamin D_3_ (25OHD_3_) is considered to be the main transport form of VD_3_ and its storage metabolite delivered to body cells via vitamin D-binding protein (VDBP) (Shymanskyi et al., 2020). 1,25(OH)_2_D_3_ can be synthesized and metabolized locally in the CNS from 25OHD_3_. This process is provided by 25-hydroxyvitamin D_3_ 1a-hydroxylase (CYP27B1), which is found in neurons and glial cells (Landel et al., 2018). Another important component of the VD_3_-auto/paracrine system is 1,25-dihydroxyvitamin D_3_ 24-hydroxylase (CYP24A1), an enzyme that catabolizes 1,25(OH)_2_D_3_ to inactive 1,24,25(OH)_3_D_3_ followed by conversion to calcitroic acid. Thus, a tight balance between the VD_3_ metabolism enzymes plays a pivotal role in maintaining an appropriate local level of 1,25(OH)_2_D_3_ for brain function. We have previously shown that chronic administration of GCs is accompanied by the development of a profound VD_3_ deficiency (Shymans’kyǐ et al., 2014). It is mainly due to impaired 25OHD_3_ synthesis in the liver as a result of a simultaneous increase in hepatocellular necrosis and caspase-3-dependent apoptosis (Lisakovska et al., 2017). However, it remains an open question to what extent VD_3_ deficiency may exacerbate glucocorticoid toxicity in the CNS.

In addition, many psychiatric and neurological diseases, including multiple sclerosis (Smolders et al., 2013), schizophrenia (Cui et al., 2021), Parkinson’s disease (Fullard and Duda, 2020), diabetic peripheral neuropathy (Xiaohua et al., 2021), and other age-related neurological outcomes (Berridge, 2017) correlate with low plasma levels of 25OHD_3_, suggesting that adequate VD_3_ levels may prevent, cure, or at least alleviate some mental disorders. Therefore, in the context of understanding the role of vitamin D_3_ in the functioning of the CNS, both under normal and diseased conditions, it is important to establish whether chronic administration of GCs can affect the circulating pool of VD_3_ and alter the ratio of the main components of the VD_3_-auto/paracrine system, as well as to what consequences these changes at the molecular, cellular, functional and behavioral levels can lead to.

Given all of the above, the aim of the study was to identify the relationship between vitamin D status and behavioral, structural, functional, and molecular changes associated with neurotoxicity caused by the synthetic glucocorticoid prednisolone in experimental rats. For the fullest characterization of vitamin D status in animals, we measured the circulating pool of VD_3_ (serum and cerebrospinal fluid (CSF) levels of 25-hydroxyvitamin D_3_), local 25OHD_3_ content in brain tissues, and the levels of major components of VD_3_-auto/paracrine system in the brain tissue—VDR, VDBP, CYP27B1, and CYP24A. To evaluate behavioral changes associated with prednisolone and vitamin D_3_ administration, we performed the forced swim test (FST), open-field test (OFT), elevated plus maze (EPM), and conditioned fear test. Histological changes were examined by staining the hippocampus, cortex, thalamus, and cerebellum with hematoxylin-eosin (H&E) or toluidine blue. At a functional level, we performed recording of an array of microelectrodes of hippocampal networks to study long-term synaptic plasticity (LTP). Depolarization-induced exocytosis in isolated nerve endings of the rat brain and the rate of fusion of synaptic vesicles with plasma membranes were also detected. To complete a comprehensive study of the mechanisms of neuroprotective action of vitamin D_3_ under glucocorticoid toxic load, we finally evaluated molecular changes at the level of GR, nuclear factor κB (NF-κB) p65 subunit and its phosphorylated form (at Ser 311), NF-κB inhibitor (IκB), poly(ADP-ribose)polymerase-1 (PARP-1), covalent protein modifications as the markers of oxidative-nitrosative and genotoxic stress (poly(ADP)-ribosylated (PAR), carbonylated and nitrated proteins) in brain tissue.

## 2. Materials and methods

### 2.1. Animals and general experimental design

**FIGURE 1:**
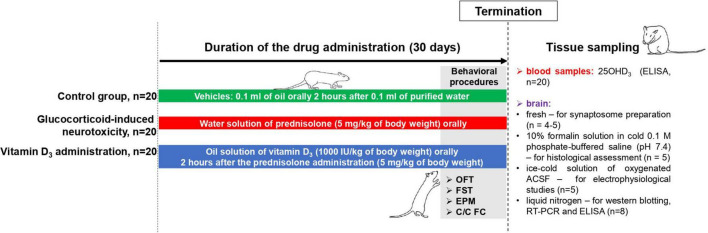
Schematic representation of the experimental design and timescale. Each experimental group included 20 animals: (1) The control group that received purified water (0.1 ml) and oil solution (0.1 ml) as vehicles; (2) the group with glucocorticoid-induced neurotoxicity, in which rats received orally the water solution of synthetic glucocorticoid prednisolone (Biopharma, Ukraine) at a dose of 5 mg/kg of body weight (30 days) and additionally oil solution (0.1 ml) as vehicle; (3) the group that received the oil solution of vitamin D_3_ (Sigma-Aldrich, USA, C9756) at a dose of 1,000 IU/kg of body weight *per os* (30 days) on the background of prednisolone administration (5 mg/kg of body weight). Behavioral procedures (OFT, open-field test; FST, forced swim test; EPM, elevated plus maze; C/C FC, contextual/cued fear conditioning test) were performed during 3 days before the decapitation with the concurrent drug administration (*n* = 8 per group). At the end of the experimental treatment, the rats were anesthetized by an intraperitoneal injection of chloral hydrate (40 mg/100 g of body weight), decapitated with a guillotine. Blood samples were taken from the inferior *vena cava* to measure 25OHD_3_ by ELISA (*n* = 20 per group). The whole brain was quickly dissected and was used fresh (for synaptosome preparation, *n* = 4–5) or transferred into the appropriate buffer depending on the procedures: 10% formalin solution in cold 0.1 M phosphate-buffered saline (pH 7.4)—for histological assessment (*n* = 5 per group); an ice-cold solution of oxygenated (95% O_2_, 5% CO_2_) artificial cerebrospinal fluid (ACSF) solution (119 mM NaCl, 2.5 mM KCl, 2.0 mM CaCl_2_, 1.3 mM MgCl_2_, 26 mM NaHCO_3_, 1.0 mM NaH_2_PO_4_, 11 mM glucose, pH 7.35)—for electrophysiological studies (*n* = 5 per group); and a liquid nitrogen—for western blotting, RT-PCR and ELISA (*n* = 8 per group).

Female Wistar rats (100 ± 5 g) were housed at the Animal Facility of the Palladin Institute of Biochemistry with free access to a standard rodent diet and drinking water *ad libitum*. Animals were kept in a temperature-controlled room and exposed to a daily 12/12 h light/dark cycle. The general scheme of animal experimental design and timescale are shown in Figure 1. After acclimatization for 1 week, the rats (*n* = 60) were randomly divided into three experimental groups: (1) the control group (*n* = 20); (2) the group that received the synthetic glucocorticoid prednisolone (5 mg/kg of body weight, *per os*, 30 days, *n* = 20); (3) the group that received prednisolone (5 mg/kg of body weight) and vitamin D_3_ (1,000 IU/kg of body weight, *per os*, 30 days, *n* = 20). Glucocorticoid prednisolone (30 mg/ml) was purchased from Biopharma (Ukraine) and was solved in the purified water for injections for further oral administration to rats. Vitamin D_3_ (cholecaciferol, Sigma-Aldrich, USA, C9756) was administered to rats as oil solution. At the end of the experimental treatment, the rats were anesthetized by intraperitoneal injection of chloral hydrate (40 mg/100 g of body weight), decapitated with a guillotine, and blood was taken from the inferior *vena cava*. The brain was quickly dissected and transferred to the appropriate buffer depending on the following procedures. All samples that were not used immediately were stored at −80°C.

### 2.2. Ethical statement

We performed all animal procedures in accordance with the protocols approved by the Animal Care Ethics Committee of the Palladin Institute of Biochemistry (Protocol #1, 26/01/2018), adopted on the basis of national and international directives and laws relating to animal welfare: European Convention for the protection of vertebrate animals used for experimental and other scientific purposes (Strasbourg, France; 1986), Bioethical expertise of preclinical and other scientific research conducted on animals (Kyiv, Ukraine; 2006).

### 2.3. Behavioral procedures

#### 2.3.1. Open-field test (OFT)

The rats were examined in a testing apparatus (1.0 × 1.0 m polypropylene box with 0.5 m-height walls) with a floor divided into 25 equal squares. The box was illuminated with white light (60 lx) during testing. Animals were individually placed in a corner of the box and allowed to freely explore the environment for 10 min. The following parameters were evaluated: the total distance traveled (mm/10 min), the time spent on 9 internal and 16 outer arena squares.

#### 2.3.2. Forced swim test (FST)

Forced swim test was performed according to the modified protocol described here (Slattery and Cryan, 2012). During the pretest (Day 1), rats were placed individually into the swimming cylinder (1.0 m height, 0.3 m diameter, with 0.5 m of warm water, +25°C) and left to swim for 15 min, then removed from water, dried with a towel and returned to the home cages. Twenty-four hours after the pre-test, the rats were individually forced to swim inside a vertical Plexiglas cylinder containing 0.5 m of water (+25°C). The camera was placed directly above the swimming cylinder and video recording was started before the rats were placed into the cylinder. After 5 min in the water, the rats were removed and allowed to dry for 20 min before being returned to their home cages. In the subsequent analysis, we defined the times for “immobile” (immobility), “mobile” (swimming), and “highly mobile” (climbing) behavior.

#### 2.3.3. Elevated plus maze (EPM)

The EPM apparatus consisted of two opposite open arms (0.5 m × 0.1 m) and two closed arms (0.5 m × 0.1 m × 0.4 m) raised to a height of 0.5 m above the floor. The junction area of the four arms (central platform) was 0.1 m × 0.1 m. Lighting in the maze was 30 lx. For the EPM test, each animal was placed on the central platform of the maze facing one of the open arms. Total distance traveled, number of open arm entries, and time spent in open arms were assessed over a 5-min period. In the EPM, anxiety levels were measured by comparing the amount of time spent in the open arms to total time.

#### 2.3.4. Contextual/Cued fear conditioning test

##### 2.3.4.1. Conditioning apparatus

A conditioning box (45 cm × 45 cm × 40 cm; custom-made) with an electric grid floor was located in a sound-attenuating chamber (50 cm × 80 cm × 60 cm) to reduce external noise and visual stimulation. After each test, the conditioning box was cleaned with water containing a small amount of neutral-smelling detergent. The behavior of the rats was recorded through the side wall of the conditioning box with a camera attached to the inner roof of the chamber. The light, tone, and foot shock in the conditioning box were controlled by the custom-made software.

##### 2.3.4.2. Contextual/Cued fear conditioning procedure

The contextual/cued fear conditioning test was performed for three consecutive days as reported previously, with some modifications (Bogovyk et al., 2017). One day prior to fear-conditioning learning (Day 1), each rat was habituated to the experimental chamber for 10 min. On Day 2 (training day), the animals were placed in the same chamber for contextual fear-conditioning training. After 60 s of free exploration, an auditory tone cue (70 dB, 10 kHz, 20 s) was presented followed by a mild foot shock (0.5 mA, 0.5 s), three tone-foot shock pairings with a 2-min interstimulus interval were applied. After the shock, the animal remained in the experimental chamber for another 60 s. On Day 3 (24 h after the previous session; testing day), the rats were subjected to a similar procedure, except that the foot shock was not given. Duration of freezing (no movement for at least 1 s) was measured during the last 20 s before the onset of an auditory tone (contextual fear conditioning) and during the tone (cued fear conditioning) twice, on the training and testing days. Spontaneous freezing refers to freezing within 20 s before the onset of an auditory tone during the training session.

### 2.4. Histological assessment of the structure of hippocampus, prefrontal cortex, cerebellum, and thalamus

For histological examination, rat brains were fixed in 10% formalin solution in cold 0.1 M phosphate-buffered saline (PBS, pH 7.4), dehydrated in increasing concentrations of ethanol, and embedded in paraffin (Leica-Paraplast Regular, 39601006, Leica Biosystems Inc., USA) according to the standard procedure. Paraffin sections 5 μm thick were prepared on a Thermo Microm HM 360 Rotary microtome (Microm GmbH, Germany). Sections were deparaffined in xylene and then rehydrated in decreasing concentrations of ethanol (100%, 95%, 80% and 70%) and distilled water, followed by staining for 5 min with 0.05% toluidine blue (pH 4.5) using the Nissl technique (García-Cabezas et al., 2016) or hematoxylin and eosin (H&E; 3 min for hematoxylin and 45 s for eosin). Ten sections from hippocampus, prefrontal cortex, cerebellum, and thalamus of each animal were taken in a systematic randomized manner and covered with Histofluid coverslips (6900002, Paul Marienfeld EN, Germany). The sections were examined under a light microscope (Olympus BX 51, Japan) at ×400 magnification. Digital photographs were taken using CarlZeiss software (AxioVision SE64 Rel.4.9.1). The cells with morphological signs of cytolysis, hyperchromatosis, karyolysis, and karyopyknosis were considered affected.

### 2.5. Synaptosome preparation and isolation of synaptic vesicles from rat brain

Purified synaptosomes were obtained from Wistar rat brains by the differential centrifugation (Kasatkina et al., 2018) using Ficoll-400 density gradient centrifugation of crude samples. Synaptosomes were used within 2–4 h after isolation.

Synaptic vesicles (SVs) were isolated from the cerebral hemispheres according to the following method (De Lorenzo and Freedman, 1978). Crude (unpurified) synaptosomes were lysed with a buffer containing 1 mM EGTA, 10 mM Tris-HCl, pH 8.1 (3 ml/g of brain tissue) and then incubated at +4°C for 60 min. The samples were centrifuged at 20,000 *g* for 30 min, followed by the next supernatant centrifugation step at 55,000 *g* for 60 min. The last centrifugation step was carried out at 130,000 *g* (+4°C, 60 min) to obtain the SVs fraction (pellet) and the synaptosomal fraction of cytosolic proteins (supernatant). The SV pellet was resuspended in 10 mM Tris-HCl (pH 7.5). The purity of the SV fraction was assessed by measuring the activity of Na^+^/K^+^-ATPase, a plasma membrane marker that was not detected in the purified SV fraction.

### 2.6. Depolarization-induced exocytosis from isolated nerve terminals

The isolated synaptic terminals (synaptosomes) were preincubated at +37°C for 10 min and fluorescence (F) was measured on a Hitachi 650-10S spectrofluorimeter (Japan) at excitation and emission wavelengths of 490 and 530 nm (2 nm slit), respectively. High K^+^ (35 mM) depolarization of the plasma membrane was achieved in Ca^2+^-containing medium at the steady-state of acridine orange (AO) fluorescence. The traces were normalized to the similar data in the absence of synaptosomes:


$$F=F_{t}/F_{0},$$

where *F*_0_ is the fluorescence intensity measured upon AO quenching in the solution, and *F*_*t*_ is the fluorescence intensity of AO in the presence of synaptosomes.

### 2.7. Synaptic vesicle fusion with plasma membranes in cell-free system

We employed the octadecyl rhodamine B chloride (R18) technique to detect SV fusion with target synaptic plasma membranes. The fusion was recorded on a Hitachi 650-10S spectrofluorimeter (Japan) in a suspension of R18-labeled SVs (1 nmol/240 μg of synaptosomal protein) and unlabeled plasma membranes in a ratio of 1:8 (according to protein concentration) after addition of Ca^2+^ (Trikash et al., 2010). An increase in the fluorescence signal was recorded as a result of membrane fusion and R18 dequenching. To quantify the probe dilution during the fusion of vesicles with target membranes, the initial fluorescence signal of R18-labeled SVs (I_*o*_) was measured. The time-dependent dequenching of the fluorescence signal (I) was recorded within 4 min after the start of testing. At the end of each experiment, C_12_E_8_ detergent was added to the reaction mixture at a final concentration of 0.1%, at which the maximum value (Id) of the dequenched fluorophore signal was obtained. The fusion rate at any given time is proportional to the percentage of fluorescence dequenching (%FD) and thus can be calculated as:


$$\%\text{FD}=100\left(\text{I}-{\text{I}}_{\text{o}}\right)/\left({\text{I}}_{\text{d}}-{\text{I}}_{\text{o}}\right)$$

### 2.8. Electrophysiology

#### 2.8.1. Isolation of the rat hippocampal sections and slice preparation

For electrophysiological studies, experimental rats were deeply anesthetized with diethyl ether followed by decapitation. The brain was carefully removed from the skull and placed in a Petri dish filled with an ice-cold solution of oxygenated (95% O_2_, 5% CO_2_) artificial cerebrospinal fluid (ACSF) solution (119 mM NaCl, 2.5 mM KCl, 2.0 mM CaCl_2_, 1.3 mM MgCl_2_, 26 mM NaHCO_3_, 1.0 mM NaH_2_PO_4_, 11 mM glucose, pH 7.35). The cerebellum was dissected with a surgical blade and a median sagittal incision was made separating the cerebral hemispheres. The hippocampus was carefully separated from the surrounding tissues and placed in a pre-prepared agar block (3.7% agar). The agar blocks with hippocampi were glued to the surface of a vibrotome plate (SYS-NVLS, World Precision Instr., USA) to ensure its reliable fixation during sectioning. The slice thickness was 400 μm. After preparation, hippocampal sections were placed in an incubation chamber with oxygenated ACSF (+26^°^C) for 1.5–2 h.

#### 2.8.2. Electrophysiological studies

Extracellular recordings were performed on freshly isolated slices of the hippocampus. The slices were transferred to a submersion-type thermostatic chamber (Warner Instrument Corp., USA), where the sections were continuously perfused with oxygenated ACSF at a rate of 2–4 ml/min (+30–32°C). The stimulating and recording electrodes were placed on the slice surface at a distance of approximately 400 μm from each other. The synaptic response was induced by stimulating the S**c**haffer collaterals using a concentric bipolar stimulating electrode and an isolated stimulation unit (ISO-Flex, AMPI, Israel). Recording of the extracellular field potential (fEPSP) was performed from the hippocampal CA1 *striatum radiatum* layer using glass microelectrodes (resistance of 1–3 MΩ when filled with ACSF) and a patch-clamp amplifier (RK-400, Bio-Logic Science Instruments, Grenoble, France). Recordings were digitized using an analog-to-digital converter (NI PCI-6221, National instruments, Austin, TX, USA) and stored on a computer running WinWCP software (Strathclyde Electrophysiology Software, University of Strathclyde, Glasgow, UK). For the baseline recording (0.33 Hz for 10 min), the stimulus intensity was set to elicit field excitatory postsynaptic potential (fEPSP) that was approximately 30–40% of the maximum response. To induce long-term plasticity (LTP), high-frequency tetanic stimulation (HFS) of 100 pulses at a frequency of 100 Hz was delivered at a baseline stimulation intensity. LTP was assessed by averaging the synaptic response from 30 to 40 min after HFS. LTP studies were carried out on 12 slices (7 animals) in the control group, 12 slices (6 animals) in the prednisolone-supplemented group, and 12 slices (5 animals) in the VD_3_-supplemented group. No more than three slices per animal were used. Offline data analysis was performed using Prism 6 (GraphPad, La Jolla, CA, USA), and Origin 7.5 (OriginLab, Northampton, MA, USA) software. Statistical comparison of changes in synaptic response was performed by measuring the initial slope of fEPSP. For LTP analysis, data from each experiment were normalized to baseline.

### 2.9. RNA isolation and real-time PCR

Total RNA was isolated from rat brain using the innuPREP RNA Mini Kit (Analytik Jena AG, Germany). The mRNA concentration was determined on a DS-11 Spectrophotometer/Fluorometer (DeNovix, USA). The Maxima H Minus First Strand cDNA Synthesis Kit (Thermo Fisher Scientific Inc., USA) was used to synthesize cDNA samples for subsequent RT-PCR on a Standard real-time PCR Thermal Cycler (Analytik Jena AG, Germany). Specific primer sequences for *Vdr*, *Vdbp*, *Cyp27b1, Cyp24a1, Nf*-κ*b*, *I*κ*b-alpha*, and the glyceraldehyde 3-phosphate dehydrogenase (*Gapdh*) reference gene were designed using Primer BLAST software and used at a working concentration of 10 μM:

**Table:** 

Gene	Forward 5′→3′	Reverse 5′→3′
*Vdr*	TCATCCCTACTGTGTCCCGT	TGAGTGCTCCTTGGTTCGTG’
*Vdbp*	AAACCCTTAGGGAATGCTGC	TTTTGTCCTCAGTCGTTCCG
*Cyp27b1*	TGGGTGCTGGGAACTAACCC	TCGCAGACTGATTCCACCTC
*Cyp24a1*	TTCGCTCATCTCCCATTCGG	TTGCTGGTCTTGATTGGGGT
*Gapdh*	TGAACGGGAAGCTCACTGG	TCCACCACCCTGTTGCTGTA
*Nf*-κ*b*	GTACTTGCCAGACACAGACGA	CTCGGGAAGGCACAGCAATA
*I*κ*b-alpha*	TGAAGTGTGGGGCTGATGTC	AGGGCAACTCATCTTCCGTG

Target genes were amplified for 60 cycles using Maxima SYBER Green/ROX qPCR Master Mix (Thermo Fisher Scientific Inc., USA). Relative mRNA expression calculations were performed according to the 2^–ΔΔ*Ct*^ comparison method. The expression level of each gene was normalized for GAPDH in the same samples and then calculated as a fold change compared to the control.

### 2.10. Western blot analysis

Using Western blotting, we measured the following protein levels in total brain tissue lysates. First, VDR was examined to evaluate the effect of chronic administration of prednisolone on the main component of the vitamin D auto/paracrine system. 3-Nitrotyrosine, carbonylated and poly(ADP)-ribosylated proteins (PAR), as well as PARP-1 attracted our attention as reliable markers of oxidative-nitrosative and genotoxic stress in brain tissue. Finally, the glucocorticoid receptor (GR), NF-κB p65 phosphorylated at Ser311, and IκB were measured to characterize the relationship of VDR and GR with NF-κB transcriptional activity. Total protein extracts were prepared from frozen brain tissue according to a standard protocol using RIPA buffer (20 mM Tris-HCl, pH 7.5; 150 mM NaCl; 1% Triton X-100; 1 mM EGTA; 0.1% SDS; 1% sodium deoxycholate; 10 mM sodium pyrophosphate) and a protease inhibitor cocktail (PIC, Sigma, USA), and then the samples were sonicated and centrifuged. The supernatants were stored at −80°C until required. Lysate samples containing 60 μg of protein were subjected to sodium dodecyl sulfate polyacrylamide gel electrophoresis (SDS-PAGE) using a 10–15% gel followed by protein transfer to a nitrocellulose membrane. The membrane was then blocked for 1 h with 5% skimmed milk diluted in Tris-buffered saline with Tween (TBST, 150 mM NaCl, 10 mM Tris-HCl, and 0.1% Tween; pH 7.5) and incubated overnight at +4°C with one of the following primary antibodies (VDR: 1:200, sc-13133, Santa Cruz; 3-nitrotyrosine: 1:2,500, #05-233, Merck Millipore; carbonylated proteins: 1:1,000, ab-178020, Abcam; poly(ADP)-ribosylated proteins: 1:1,000, 4335-MC-100_AC, Trevigen; PARP-1: 1:1,000, #9542, Cell Signaling; GR: 1:250, sc-1004, Santa Cruz; NF-κB p65 phosphorylated at Ser 311: 1:200, sc-33039, Santa Cruz; and IκB-α: 1:1,000, sc-847, Santa Cruz). Subsequently, the membrane was thoroughly rinsed and incubated for 1 h with secondary antibodies: anti-rabbit IgG (H + L)-HRP conjugate (1:4,000, #1721019, Bio-Rad) or anti-mouse IgG (Fab Specific)-Peroxidase (1:2,500, A9917, Sigma) at room temperature. Thereafter, the membrane was developed with chemiluminescent agents: p-coumaric acid (Sigma, USA) and luminol (AppliChem GmbH, Germany). Target protein immunoreactive signals were quantified by densitometry and values were adjusted for the corresponding β-actin level (1:10,000, A3854, Sigma). Immunoreactive bands were quantified using Gel-Pro Analyzer v3.1 software.

### 2.11. ELISA 25-hydroxyvitamin D_3_ assay

Using a commercial ELISA kit ≪25-OH-Vitamin D_3_≫ (Immunodiagnostics, Germany), in accordance with the manufacturer’s instructions, the VD_3_ status was assessed by determining the concentration of 25-hydroxyvitamin D_3_ in the serum, cerebrospinal fluid and brain homogenate of rats. Cerebrospinal fluid was obtained by puncture of the atlanto-occipital space. Brain homogenates were prepared from frozen brain tissue (100 mg) that was ground in a porcelain mortar with liquid nitrogen, lysed in PBS with 1% Tween-20 (pH 7.4), then sonicated and incubated on ice for 2 h. Next, the samples were centrifuged at 14,000 *g* for 20 min at +4°C. The supernatant was carefully collected and aliquots were stored at −20°C avoiding thawing/freezing.

### 2.12. Statistics

The results of all experiments were expressed as the mean ± standard deviation for at least seven rats per group. The hypothesis about the normality of data distribution was tested using the Shapiro–Wilk test. Differences between means were estimated using the ANOVA followed by Tukey’s *post hoc* test. To compare difference between groups in neuronal morphometric parameters, statistical significance was determined using a two-tailed unpaired Student’s *t*-test. For behavioral data, the non-parametric Kruskal–Wallis test was used, if necessary, followed by Dunn’s *post hoc* test. The significance criterion was *p* ≤ 0.05. Statistical analysis was performed using Origin Pro 8.5 software (OriginLab Corporation, Northampton, MA, USA). For behavioral datasets, statistical significance was analyzed using the Origin 9 and Graph Pad Prism 6 software.

## 3. Results

### 3.1. Effect of prednisolone and vitamin D_3_ on brain-to-body weight ratio

The brain-to-body weight ratio is commonly used in neurotoxicity studies to estimate the presence of general morphopathological changes in the brain. As can be seen from Table 1, the administration of prednisolone slowed down the growth of rats and reduced the average body weight by 70.65% compared with the control group (Tukey’s test, *p* = 0.000051), while there was no significant difference in the average brain mass between the two groups (Table 1, Tukey’s test, *p* > 0.05). An assessment of the brain-to-body weight ratio revealed that GC slightly increased it by 29.04% compared with the control group (Tukey’s test, *p* = 0.0031). Treatment with VD_3_ enhanced mean body weight by 15.97% compared with the prednisolone group (Tukey’s test, *p* = 0.033), however, it did not lead to any ameliorative effect on the brain-to-body weight ratio compared with the prednisolone group (Tukey’s test, *p* > 0.05).

**TABLE 1:** Effect of prednisolone and vitamin D_3_ administration on brain-to-body weight ratios in rats (M ± m, *n* = 20).

Groups	Control	Prednisolone	Prednisolone + vitamin D_3_
Terminal body weight (g)	241.90 ± 9.16	170.90 ± 6.64*	198.20 ± 5.39*^#^
Brain weight (g)	1.676 ± 0.054	1.535 ± 0.057	1.637 ± 0.052
Brain-to-body weight ratio (%)	0.699 ± 0.031	0.903 ± 0.026*	0.828 ± 0.022*

*p < 0.05 vs. control.

^#^*p* < 0.05 vs. prednisolone administration.

### 3.2. Vitamin D_3_ status and the state of VD_3_-auto/paracrine system in brain after prednisolone administration and effect of vitamin D_3_ treatment

**FIGURE 2:**
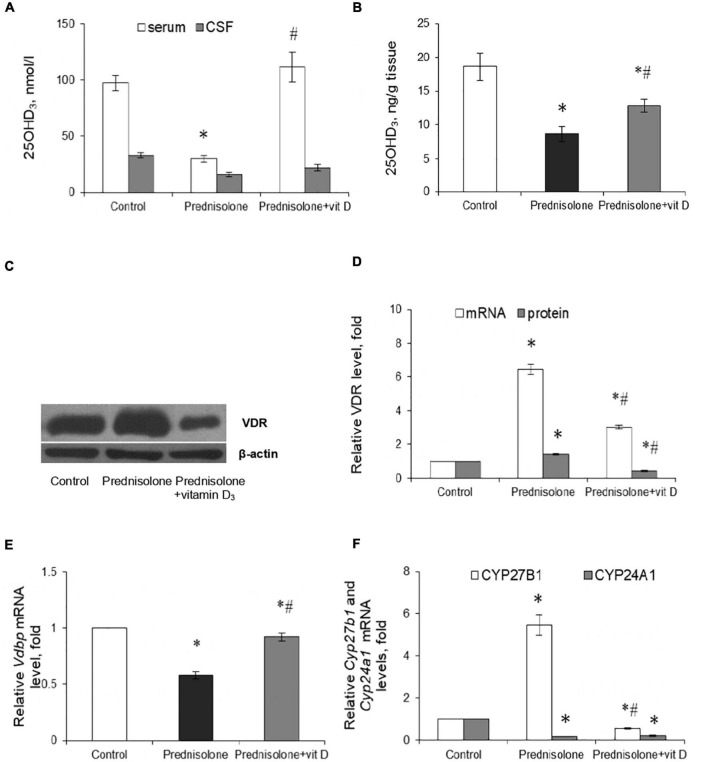
Vitamin D_3_ circulating pool and the state of vitamin D_3_-auto/paracrine system in the brain tissue. 25OHD_3_ concentration in the serum, cerebrospinal fluid **(A)** and brain homogenates **(B)** were measured by ELISA (*n* = 20). VDR protein **(C,D)** and mRNA **(D)** levels were determined by western blotting and quantitative RT-PCR respectively in rat brain tissue of three animal groups: 1—control; 2—prednisolone administration; 3—prednisolone and vitamin D_3_ administration (*n* = 8 rats/group). Representative immunoblots are shown near the bar charts **(C)**. *Cyp27b1* **(E)** and *Cyp24a1* **(F)** mRNA levels were assessed by quantitative RT-PCR. All protein levels were normalized to β-actin and mRNA levels—to *Gapdh* expression. All data are presented as mean ± SD of three independent experiments done in triplicate; *p < 0.05 denotes significance compared with control, ^#^*p* < 0.05 denotes significance compared with prednisolone administration.

In the context of the essential role of vitamin D_3_ for the CNS functioning, the question arose whether chronic GC treatment could affect the circulating VD_3_ pool and alter the content of the main components of the VD_3_-auto/paracrine system. First, we measured the level of 25-hydroxyvitamin D_3_ as a reliable marker of VD_3_ bioavailability in serum, cerebrospinal fluid, and brain homogenates. Figure 2A shows a pronounced drop in 25OHD_3_ in the serum of rats supplemented with GC (by 3.24-fold, up to 30.00 ± 3.04 nmol/L) compared with the control animals (97.23 ± 7.01 nmol/L, Tukey’s test, *p* = 0.000001). In the CSF, we observed half the content of 25OHD_3_ in the prednisolone group (16.00 ± 2.05 nmol/L) than in the control group (33.00 ± 2.50 nmol/L, Figure 2A). Finally, depletion of the circulating VD_3_ pool was correlated with a significant decrease in the 25OHD_3_ level in the brain tissue extracts, indicating impaired bioavailability of the VD_3_-prohormone in the nervous tissue. Figure 2B demonstrates that the 25OHD_3_ level after prednisolone supplementation was 8.64 ng/g brain tissue compared to 18.60 ng/g brain tissue in the control animals, representing a 2.15-fold decrease (Tukey’s test, *p* = 0.022).

Vitamin D_3_ supplementation caused a partial restoration of the 25OHD_3_ content. To a greater extent, the normalizing effect concerned the serum, where the 25OHD_3_ level rose to 111.43 ± 13.17 nmol/L and there was no significant difference with the control group. In the CSF, we also observed a tendency to restore the level of 25OHD_3_, the value of which reached 22 ± 3.1 nmol/L. This is 37.5% higher than in the prednisolone group. The 25OHD_3_ level also increased in the brain extract to 12.88 ± 0.9 ng/g of tissue, which was 49% higher than in the prednisolone group (Tukey’s test, *p* = 0.042), but remained still lower (by 44.4%) than in the control. Collectively, these data indicate a profound GC-induced VD_3_ deficiency and depletion of the circulating VD_3_ pool under the influence of prednisolone, however, vitamin D_3_ treatment may partially restore the circulating VD_3_ pool, suggesting that this abnormality is reversible.

Since the hormonally active form of VD_3_ in different cell types exerts its biological action through specific receptors for 1,25(OH)_2_D_3_ – VDR, we also examined the VDR expression in the brain at the transcriptional and translational levels (Figures 2C, D). It was shown that after prednisolone administration, the level of *Vdr* mRNA in the brain increased by 6.45 times (Tukey’s test, *p* = 0.000003) (Figure 2D). We further confirmed an increase in VDR protein by western blot (Figures 2C, D), which was 41% higher than in the control group (Tukey’s test, *p* = 0.005). In turn, a strong reducing effect of VD_3_ on the expression of *Vdr* mRNA (by 2.14-fold, Tukey’s test, *p* = 0.000001) and protein (by 3.44-fold, Tukey’s test, *p* = 0.000002) was found compared with the prednisolone group.

Despite the observed disturbances in the VDR expression, the next question arises whether prednisolone is able to affect the VD_3_ transport. VDBP is considered to be the major plasma transporter for all VD_3_ metabolites; however, its function in the brain is not fully understood. Therefore, we examined the level of *Vdbp* mRNA and found that prednisolone caused a 42%-lowering effect (Tukey’s test, *p* = 0.002) compared to the control, while VD_3_ supplementation restored its level to the control value (Figure 2E).

To complete this part of the study, we focused on enzymes involved in VD_3_ metabolism and considered important factors that contribute to the proper functioning of the VD_3_-auto/paracrine system. As presented in Figure 2F, prednisolone induced a significant (5.46-fold, Tukey’s test, *p* = 0.00002) increase in *Cyp27b1* mRNA expression in the brain compared with the control animals, which may reflect a compensatory response of the VD_3_-auto/paracrine system to the lack of circulatory and brain levels of 25OHD_3_. Vitamin D_3_ treatment reduced *Cyp27b1* gene expression to a level that was 9.72-fold lower than in the prednisolone group (Tukey’s test, *p* = 0.00001) and 1.79-fold lower than in the control group (Tukey’s test, *p* = 0.0029).

Another component of the VD_3_-auto/paracrine system that we examined was CYP24A1, that catalyzes the inactivation of both 25OHD_3_ and 1,25(OH)_2_D_3_ (Hamamoto et al., 2006). In contrast to the effect of prednisolone on *Cyp27b1*, this glucocorticoid caused a 5.41-fold decrease in *Cyp24a1* mRNA level compared with the control group (Tukey’s test, *p* = 0.0005), while VD_3_ did not significantly affect the level of *Cyp24A1* mRNA compared with the prednisolone group (Tukey’s test, *p* > 0.05, Figure 2F).

In general, our results suggest that, despite the development of VD_3_-deficiency, prednisolone elevated, most likely compensatory, the expression of such important components of the VD_3_-auto/paracrine system as CYP27B1 and VDR, while the levels of mRNA transcripts of CYP24A1 and VDBP genes decreased in the brain, thus indicating an impairment of VD_3_ transport and catabolism. Administration of cholecalciferol restored the circulating 25OHD_3_ pool and normalized, at least in part, the levels of key components of the VD_3_-auto/paracrine system in the brain, although VD_3_ 24-hydroxylase remained unchanged.

### 3.3. Effects of prednisolone and vitamin D_3_ on depressive-like behavior, locomotion and cognitive performance

**FIGURE 3:**
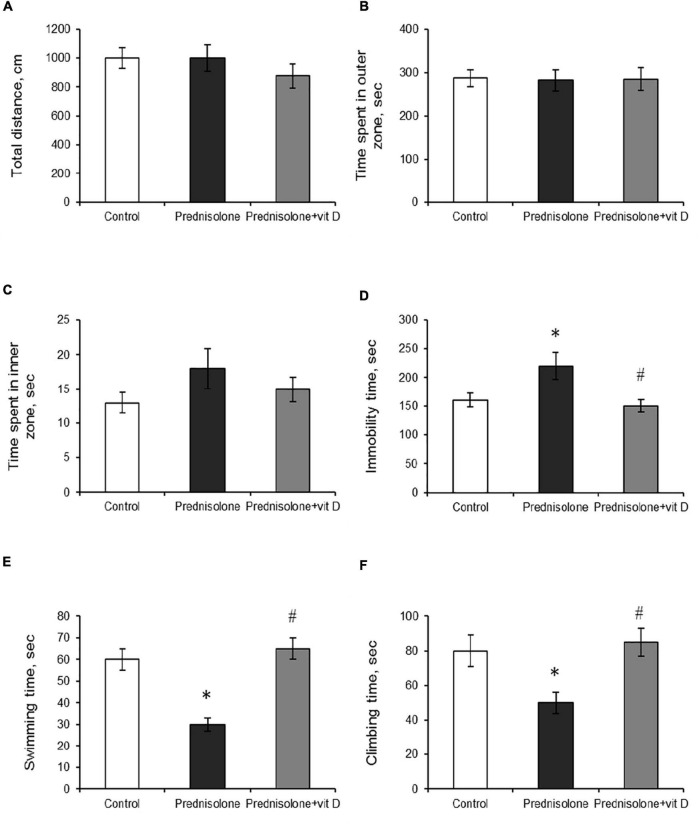
The influence of prednisolone and vitamin D_3_ on the parameters of the open field (OFT) and forced swim (FST) tests. Animals from three experimental groups: 1—control; 2—prednisolone administration; 3—prednisolone and vitamin D_3_ administration (*n* = 8 rats/group) were subjected to the OFT **(A–C)** and FST **(D–F)**. Total distance traveled **(A)**, time spent in outer **(B)** and inner **(C)** zones were calculated during the OFT. Total immobility time **(D)** and the parameters of active behavior: swimming **(E)**, and climbing **(F)** were assessed during the FST. All data are presented as mean ± SD of three independent experiments done in triplicate; *p < 0.05 denotes significance compared with control, ^#^*p* < 0.05 denotes significance compared with prednisolone administration.

To support the concept that VD_3_ deficiency may be associated with behavioral abnormalities (Schoenrock and Tarantino, 2016), we further estimated prednisolone-induced behavioral changes in animals that we found to exhibit significantly reduced VD_3_ bioavailability. The open-field test allows to examine motor function and exploratory behavior by measuring spontaneous activity in the OFT box. Animals of all groups showed no significant difference in walking distance (*p* > 0.05) and in time spent exploring 9 inner squares and 16 outer squares (*p* > 0.05), as presented in Figures 3A–C respectively.

The forced swim test has been used to assess the presence of depression-related behaviors (Slattery and Cryan, 2012). Prednisolone-administered rats demonstrated an increase in immobility time (220 ± 15 s, Figure 3D) and a decrease in active behavior (swimming 30 ± 3 s, Figure 3E, climbing 50 ± 6 s, Figure 3F) compared with the control rats (160 ± 12 s, 60 ± 5 s, 80 ± 9 s, respectively), indicating depressive-like changes. VD_3_ exerted antidepressive-like action, diminishing immobility time (150 ± 11 s) and stimulating active behavior (swimming 65 ± 5 s, climbing 85 ± 8 s) compared with the GC action.

**FIGURE 4:**
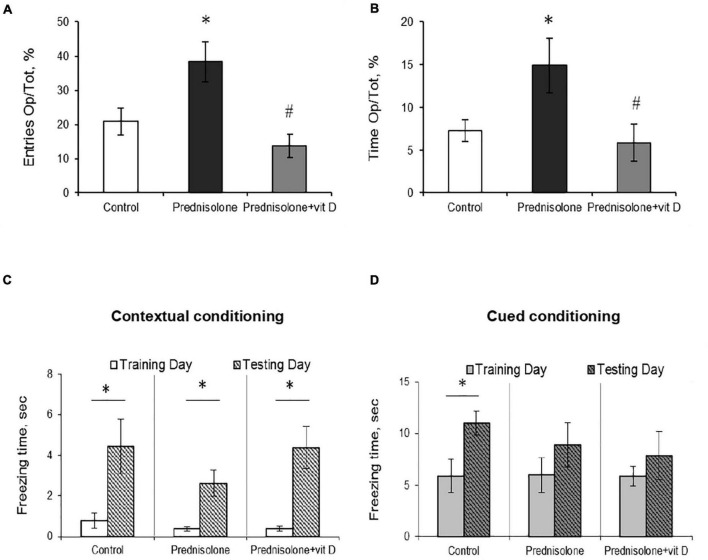
The effect of prednisolone and vitamin D_3_ on the behavior in the elevated plus maze (EPM) and contextual and cued fear conditioning and memory test. Animals from three experimental groups: 1—control; 2—prednisolone administration; 3—prednisolone and vitamin D_3_ administration (*n* = 8 rats/group) were subjected to the EPM **(A,B)** and contextual/cued conditioning test **(C,D)**. The percentage of entries into the open arms to the total time (Op/Tot) **(A)** and the ratio of time spent in the open arms to the total time (Op/Tot) **(B)** were measured during the EPM. Mean freezing time levels during the contextual **(C)** and cued **(D)** conditioning test were assessed after prednisolone and vitamin D_3_ treatment during training and testing days. All data are presented as mean ± SD of three independent experiments done in triplicate; *p < 0.05 denotes significance compared with control, ^#^*p* < 0.05 denotes significance compared with prednisolone administration.

The next task was to determine the level of anxiety in the elevated plus maze test by measuring the amount of time spent in open/closed arms. Behavior in performing this task (activity in the open arms) reflects a conflict between rodent’s preference for protected areas (closed arms) and their innate motivation to explore novel environments. In the EPM test, we observed a tendency to an increased percentage of entries into the open arms (38.33 ± 5.9%, Figure 4A) and time spent in the open arms (14.92 ± 3.16%, Figure 4B) after prednisolone administration compared with the control group (21.03 ± 3.9% and 7.3 ± 1.27%, respectively). Unexpectedly, these results showed that prednisolone did not cause the anxiety-related behavioral changes. Increased time spent in the open arms and percentage of entries are conventionally interpreted as decreased anxiety-like behavior (Shoji and Miyakawa, 2021), however, another possible interpretation can be considered: an open arm study may reflect heightened anxiety and panic response to a novel situation under certain conditions. VD_3_ administration reduced the percentage of entries into the open arms (13.88 ± 3.4%, *p* = 0.0272) and time spent in the open arms (5.92 ± 2.18%, *p* = 0.0306) compared to the prednisolone-administered group. In addition, there was no meaningful difference when comparing the EPM parameters of VD_3_-treated rats with the control group and the prednisolone group. Interestingly, time spent in the open arms of the EPM was positively correlated with immobility time in the FST (*r* = 0.296, *p* < 0.05) and negatively correlated with active behavior (*r* = −0.382, *p* < 0.01 for swimming).

After completion of the OFT, FST, and EPM tests, the rats were subjected to a contextual/cued fear conditioning test to assess an associative fear learning and memory. As shown in Figures 4C, D, there was no statistically significant difference in spontaneous freezing between all groups during the training day in the novel context (*p* = 0.65, Figure 4C) and after an auditory tone cue (*p* = 0.99, Figure 4D). We also did not observe significant differences between groups in the level of spontaneous freezing during the day of testing in both contextual and cued conditioning. However, we noted a tendency that the time of contextual conditioning freezing was significantly shorter in prednisolone-administered rats (2.63 ± 0.64 s vs. 4.44 ± 1.33 s in the control). A separate analysis of freezing time between each group revealed the statistically significant differences between training and testing days for three groups in contextual conditioning (Figures 4C, *p* = 0.049). However, no valuable difference was observed in cued conditioning freezing between all 3 groups (Figures 4D, *p* = 0.068).

Thus, we did not find any significant effects on locomotor activity or memory parameters (based on OFT and contextual/cued conditioning), while FST and EPM tests reflect the behavioral changes following prednisolone administration. In particular, FST demonstrated an increase in immobility time and a decrease in active behavior in glucocorticoid-treated rats, indicative of depressive-like changes. At the same time, the EPM results may point to the stimulation of anti-anxiety behavior induced by prednisolone; however, taking into consideration an increased time of immobility in the FST, we are more inclined to believe that the data obtained could reflect a panic-like reaction. Vitamin D_3_ treatment diminished the immobility time and stimulated active behavior, suggesting anti-depressive effect of cholecalciferol. Exploratory behavior in the EPM was restored to control levels after VD_3_ action.

### 3.4. Prednisolone-induced histological alterations of different brain regions and effect of vitamin D_3_ in their prevention

Because lesions in the hippocampus are known to be capable of disrupting contextual fear conditioning (Chen et al., 1996), and given the observed GC-induced tendency to reduce freezing time in contextual conditioning, we first assessed the presence of histopathological changes in the hippocampus. For this purpose, histological staining with toluidine blue was carried out, followed by morphometric and statistical evaluation of the CA1–CA3 regions.

**FIGURE 5:**
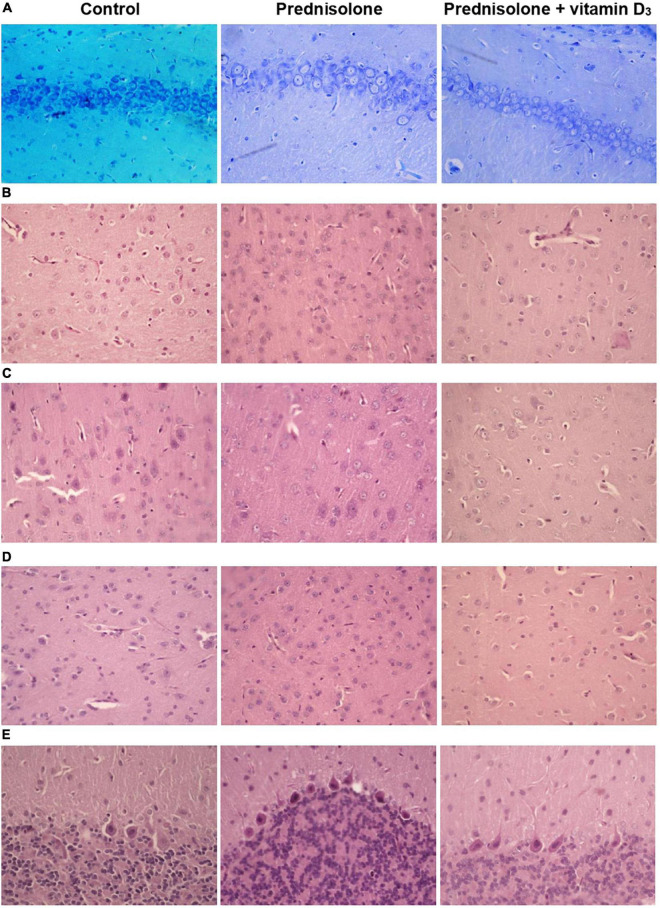
Histological examination of hippocampus, cerebral cortex, thalamus and cerebellum after prednisolone and vitamin D_3_ treatment (toluidine blue or hematoxylin & eosin staining). Representative images of toluidine blue-stained 6-μm rat CA1 hippocampal sections section **(A)**, H&E-stained cerebral cortex (prefrontal cortex) **(B)**, sensory-motor cortex **(C)**, H&E-stained thalamus sections (posterior thalamic nucleus) **(D)**, and H&E-stained cerebellar cortex **(E)** from the control, prednisolone-administered and prednisolone plus vitamin D_3_-administered rats (*n* = 5 rats/group, 400× magnification).

Histological examination revealed no visible signs of brain damage (neither hydropic neuronal dystrophy nor neurodegeneration) in all three groups (Figure 5A). Analysis of the morphometric parameters of pyramidal neurons in fields CA1–CA3 confirmed the presence of the following changes (Table 2). The perikaryon area increased by 55.1% in CA1, by 67.6% in CA2, and by 29.0% in the CA3 region in the prednisolone group compared with the control group (Student’s *t*-test, *p* < 0.001). In addition, after prednisolone administration, we observed intact, but at the same time, enlarged neuronal nuclei (by 62.55, 83.45, and 49.27% in CA1, CA2, and CA3, respectively) compared with the control (Student’s *t*-test, *p* < 0.001). The nuclei contained increased levels of euchromatin, indicating the activation of biosynthetic processes in neurons. Prednisolone also caused a slight decreasing effect on cell density in the CA1–CA3 hippocampal fields (Figure 5A). Vitamin D_3_ administration led to a significant decrease in the mean areas of both the perikaryon and the nucleus in CA1 compared with the prednisolone group (by 68.12 and 73.79%, respectively, Student’s *t*-test, *p* < 0.001), while only a slight tendency to a reduction of these parameters in CA2 and CA3 sections was found.

**TABLE 2:** Morphometric parameters of hippocampal neurons of CA1-CA3 fields (M ± m, *n* = 5).

Groups	Hippocampal subfields					
	**CA1**		**CA2**		**CA3**	
	**Mean perikaryon area, μm^2^**	**Mean area of the neuron nucleus, μm^2^**	**Mean perikaryon area, μm^2^**	**Mean area of the neuron nucleus, μm^2^**	**Mean perikaryon area, μm^2^**	**Mean area of the neuron nucleus, μm^2^**
Control	371.0 ± 11.1	186.1 ± 7.2	421.8 ± 10.0	196.4 ± 7.9	429.1 ± 8.3	218.6 ± 8.6
Prednisolone	575.4 ± 19.0*	302.5 ± 13.2*	707.1 ± 21.7*	360.3 ± 6.1*	553.6 ± 31.4*	326.3 ± 18.7*
Prednisolone + vitamin D_3_	392.0 ± 9.8^#^	223.2 ± 6.6*^#^	646.9 ± 34.0*^#^	296.7 ± 18.4*^#^	531.7 ± 18.3*	270.4 ± 8.0*^#^

*p < 0.001 vs. control.

^#^*p* < 0.001 vs. prednisolone administration.

Histological sections of the cerebral cortex demonstrate the preservation of the general morphological structure of the prefrontal and sensorimotor cortex (Figures 5B, C). In all studied samples, pyramidal neurons and glial cells were detected in all layers of the cerebral cortex, except for the molecular layer. Structurally intact microvessels, mostly hemocapillaries, were also present. We did not reveal any signs of inflammation, leukocyte infiltration, necrosis or hemorrhage (Figures 5B, C). However, in the groups of prednisolone and VD_3_, clarification of the cytoplasm was observed in large pyramidal neurons, especially in the ganglionic cell layer, probably indicating impaired functional activity of cells, inhibition of synthetic processes, and disintegration of organelles of the protein-synthesizing apparatus of neurons. We did not detect a statistically significant difference in the number of neurons in the prefrontal and sensorimotor cortex between the studied groups (Table 3). When comparing the mean area of neurons in the ganglionic layer of the cerebral cortex, there was a tendency to its increase in the prefrontal cortex after prednisolone administration compared with the control (by 13.4%, Student’s *t*-test, *p* = 0.04). In the sensorimotor cortex, a trend toward an increase in the mean area of neurons was found after VD_3_ treatment compared with the control (by 18.1%, Student’s *t*-test, *p* = 0.03) and prednisolone (by 24.1%, Student’s *t*-test, *p* = 0.035) groups.

**TABLE 3:** Morphometric parameters of the cerebral cortex (test area 550 μm × 215 μm, M ± m, *n* = 5).

Groups	Number of neurons, units per 1 test area	Mean area of the neurons of ganglionic layer of cerebral cortex, μm^2^
**Prefrontal cortex**		
Control	57.0 ± 5.8	362.2 ± 14.2
Prednisolone	55.7 ± 7.4	410.7 ± 25.2*
Prednisolone + vitamin D_3_	50.7 ± 3.7	390.3 ± 21.5
**Sensory-motor cortex**		
Control	56.7 ± 2.7	487.4 ± 16.4
Prednisolone	55.0 ± 8.8	463.5 ± 40.1
Prednisolone + vitamin D_3_	51.5 ± 2.9	575.5 ± 49.6*^#^

*p < 0.05 vs. control.

^#^*p* < 0.05 vs. prednisolone administration.

Next, we studied the effect of prednisolone and cholecalciferol on the posterior thalamic nucleus. Histological evaluation did not reveal signs of dystrophic changes, necrosis or inflammation in the thalamus of all experimental groups. We also did not detect neuronal necrosis, however, single hyperchromic gliocytes and neurons were found. In general, the histological structure of the thalamic region in all the studied groups was similar (Figure 5D). These observations were confirmed by morphometric analysis, and there was no statistically significant between-group difference, as indicated in Table 4.

**TABLE 4:** Morphometric parameters of the thalamus (posterior thalamic nucleus, test area 550 μm × 215 μm, M ± m, *n* = 5).

Groups	Number of neurons, units per 1 test area	Mean area of the neurons, μm^2^
Control	33.4 ± 3.3	388.2 ± 21.4
Prednisolone	40.8 ± 3.0	417.1 ± 20.4
Prednisolone + vitamin D_3_	34.2 ± 3.2	406.9 ± 13.3

Finally, we found a number of morphological changes in the cerebellar cortex caused by the prednisolone action, with a slight ameliorative effect of VD_3_. In general, for all experimental groups the cytoarchitecture of the cerebellar cortex was well preserved; the molecular, ganglionic, and granular layers were clearly visible (Figure 5E). In the molecular layer, single nuclei of gliocytes and processes of neurons were detected, which together formed the outer layer of the cerebellar cortex. Separate piriform neurons (Purkinje cells) in the ganglionic layer were located in a one-layered manner. The granular layer was formed by numerous small granular neurons. Thus, we did not find any neurodystrophic changes in the molecular and granular layer after prednisolone administration, while in the ganglionic layer there were strained neurons with hyperchromic staining of the cytoplasm and nuclei. Although we did not observe intergroup differences in the number of neurons and density of Purkinje cells in the cerebellar cortex (Table 5), the size of strained neurons was 43.2% and 33.2% smaller in the prednisolone group (406.1 ± 14.7 μm^2^, Student’s *t*-test, *p* = 0.009) and after VD_3_ treatment (477.7 ± 27.8 μm^2^, Student’s *t*-test, *p* = 0.012), respectively, compared with the control group (714.9 ± 48.3 μm^2^). These observations indicate that neurodegenerative changes may occur under the influence of prednisolone in the cerebellar cortex.

**TABLE 5:** Morphometric parameters of the ganglionic layer of the cerebellum (test area – width of the cortex layer 500 μm, M ± m, *n* = 5).

Groups	Number of neurons, units per 1 test area	Mean area of the neurons, μm^2^
Control	8.0 ± 0.5	714.9 ± 48.3
Prednisolone	7.8 ± 0.4	406.1 ± 14.7*
Prednisolone + vitamin D_3_	9.0 ± 0.7	477.7 ± 27.8*

*p < 0.05 vs. control.

^#^*p* < 0.05 vs. prednisolone administration.

Thus, we did not establish any valuable differences in the cytoarchitectonics of the cerebral cortex and the posterior nucleus of the thalamus between the studied groups. However, GC-induced changes were found in the hippocampus and cerebellum, namely, a decrease in cell density in the hippocampal zones, as well as a decrease in the size of neurons in the cerebellar cortex. These changes in general may indicate the onset of neurodegenerative changes under the influence of prednisolone. Vitamin D_3_ partially normalized the cytoarchitecture of the hippocampus and cerebellum.

### 3.5. Assessment of synaptic function after prednisolone administration and effect of vitamin D_3_ treatment

It is most likely that despite the absence of striking prednisolone-induced histopathological changes in the brain structures they, nevertheless, can be accompanied to some extent by impaired neuronal function. The harmful effects of GC on the brain and the impact of vitamin D_3_ therapy may primarily concern some important aspects of synaptic function, such as depolarization-induced exocytosis from isolated neurons, synaptic vesicle fusion with plasma membrane of nerve terminals and changes in long-term potentiation (LTP).

**FIGURE 6:**
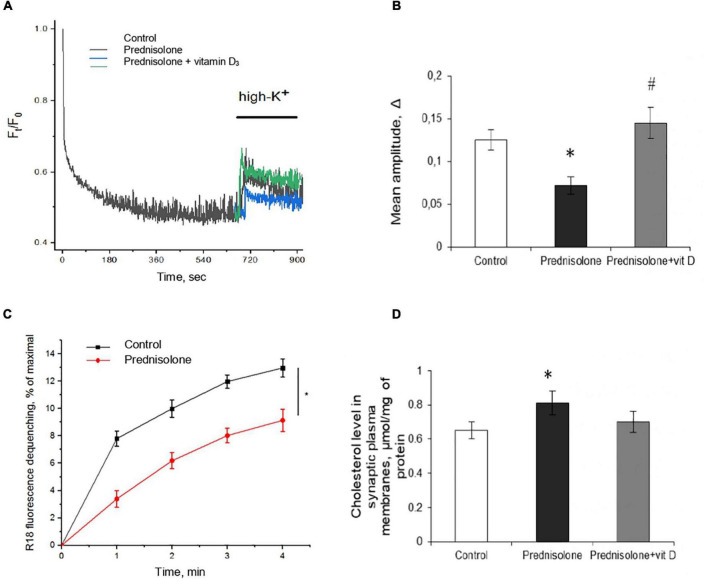
Glucocorticoid-induced disturbances of synaptic function and its correction by the vitamin D_3_ supplementation. Depolarization-induced exocytosis in rat brain nerve terminals (synaptosomes) **(A)** and quantification of the mean amplitude Δ **(B)**. Nerve terminals were isolated from the control, prednisolone-treated and prednisolone plus vitamin D_3_-treated rats. Spectrofluorimetric registration of pH-sensitive dye – acridine orange (AO), *n* = 4. The rate of synaptic vesicle fusion with plasma membranes in cell-free system **(C)**. Fusion was determined by monitoring of R18 fluorescence dequenching in suspension of R18-labeled synaptic vesicles and unlabeled synaptic plasma membranes upon Ca^2+^ application in control and prednisolone-administered rats, *n* = 5 (traces). Cholesterol content in synaptic plasma membranes **(D)** of control and prednisolone-treated rats (*n* = 5). All data are presented as mean ± SD of three independent experiments done in triplicate; *p < 0.05 denotes significance compared with control, ^#^*p* < 0.05 denotes significance compared with prednisolone administration.

First, we characterized the process of depolarization-induced exocytosis from isolated neurons and monitored vesicle-to-membrane fusion. As shown in Figures 6A, B, following high K^+^-depolarization, the nerve endings isolated from the brain of prednisolone-administered rats demonstrated a 57.5%-decrease in exocytosis amplitude compared with the control (Tukey’s test, *p* = 0.00023).

One of the reasons for the decrease in the exocytosis amplitude might be changes in the mechanisms involved in providing the fusion of synaptic vesicles with the plasma membranes of synaptic terminals. This process strictly depends on the presence of soluble cytosolic synaptic proteins and involves successive stages of interaction of synaptic vesicles with the target membrane: docking, priming, and fusion. We isolated synaptic vesicles and synaptic plasma membranes and evaluated their Ca^2+^-triggered fusion in a cell-free system. As is seen in Figure 6C, synaptic vesicles from prednisolone-administered rats show a significantly lower rate of Ca^2+^-dependent synaptic plasma membrane fusion (by 29.53%) compared to the control (Tukey’s test, *p* = 0.01).

Glucocorticoids have been reported to impair cholesterol metabolism in the brain, and its level is one of the important factors influencing the rate of synaptic vesicle fusion with each other and with the synaptic plasma membrane (Gumenyuk et al., 2013). With this in mind, we studied the content of total cholesterol in synaptic membranes isolated from the cerebral hemispheres and found an increase in its level in prednisolone-supplemented animals (0.81 ± 0.07 μmol/mg protein) compared to the control level (0.65 ± 0.05 μmol/mg protein) (Figure 6D). Elevated synaptic plasma membrane cholesterol may indicate abnormal cholesterol metabolism associated with the administration of prednisolone and is comparable to that observed in alimentary vitamin D deficiency (Kasatkina et al., 2018).

After supplementation with VD_3_, we observed an increase in mean amplitude of 103.07% compared with the prednisolone group (Tukey’s test, *p* = 0.00002, Figure 6B), which almost reached the level of control values. Recovery of depolarization-induced release of synaptic vesicles after VD_3_ treatment (Figure 6A) indicates the reversibility of prednisolone-induced changes in stimulated secretion. Contrary to our expectations, we failed to find statistically significant changes in cholesterol levels after VD_3_ supplementation compared with the prednisolone group (*p* > 0.5, Figure 6D).

**FIGURE 7:**
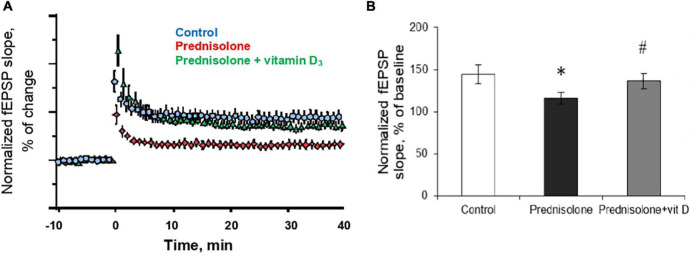
Effect of vitamin D_3_ on prednisolone-induced alteration of synaptic plasticity in shaffer-collateral—CA1 synapses. fEPSP slope recorded before and after tetanic stimulation was shown **(A)** and the normalized fEPSP amplitudes was calculated **(B)** for the control, prednisolone-treated and prednisolone plus vitamin D_3_-treated rats (*n* = 5 rats/group). All points at panel **(A)** represent the average of three consecutive responses recorded every 20 s at the indicated time points. Data are presented as mean ± SD; *p < 0.05 denotes significance compared with control, ^#^*p* < 0.05 denotes significance compared with prednisolone administration.

In the next series of experiments, we studied changes in long-term potentiation in animals in response to chronic prednisolone action and after vitamin D_3_ administration. For the LTP study, we used a common tetanic stimulation protocol, which is classic for studying plasticity in the hippocampal CA1 area (100 stimuli, at a frequency of 100 Hz). In this experiment, we used sections of the septal part of the hippocampus involved in spatial memory processes. Analysis of the LTP parameters (Figure 7A) showed a decrease in fEPSP by 16.6% after GC administration compared with the control (*p* = 0.0025; Figure 7B) that reflects an alteration of the LTP. Vitamin D_3_ treatment restored the prednisolone-induced LTP (prednisolone vs. vitamin D_3_, *p* = 0.033) to nearly control levels (VD_3_ vs. control, *p* = 0.56).

Thus, in relation to functional disorders, we have established a GC-induced decrease in the amplitude of high K^+^-stimulated exocytosis, the rate of Ca^2+^-dependent fusion of isolated synaptic vesicles with synaptic plasma membranes that may be partially associated with an increase in cholesterol levels in the latter. A reduced level of LTP was found in the synapses of the hippocampus. Taken together, all these impairments may underlie the observed changes in behavior after glucocorticoid loading. In turn, vitamin D_3_ therapy tended to restore, at least partially, the above changes, regardless insignificant effect on cholesterol levels.

### 3.6. Prednisolone-induced oxidative-nitrosative stress in brain tissue and effect of vitamin D_3_ treatment

**FIGURE 8:**
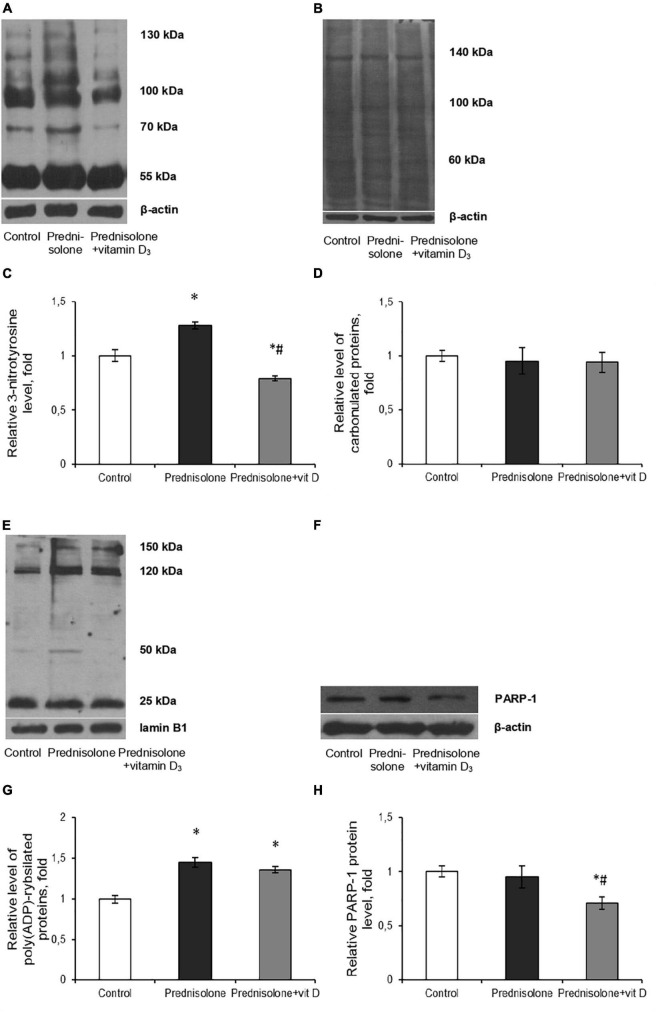
Oxidative-nitrosative stress after prednisolone and vitamin D_3_ administration. Levels of 3-nitrotyrosine **(A,C)**, carbonylated proteins **(B,D)**, poly(ADP)-rybosilated proteins **(E,G)** and PARP-1 **(F,H)** were determined by western blot analysis in rat brain tissue of three animal groups: 1—control; 2—prednisolone administration; 3—prednisolone and vitamin D_3_ administration (*n* = 8 rats/per group). Representative immunoblots are shown above the bar charts. Protein levels were normalized to β-actin and/or lamin B1. All data are presented as mean ± SD of three independent experiments done in triplicate; *p < 0.05 denotes significance compared with control, ^#^*p* < 0.05 denotes significance compared with prednisolone administration (one-way ANOVA, Tukey’s *post-hoc* test).

Excess formation of reactive oxygen/nitrogen species (ROS/RNS) is considered to be one of the universal mechanisms leading to cell injury and CNS dysfunction (Oosthuizen et al., 2005). It is known that nitric monooxide (NO) can interact with superoxide-anion radicals to form an unstable and highly reactive peroxynitrite (ONOO-). Tyrosine residues of protein molecules are sensitive targets for the action of peroxynitrite. Thus, the content of 3-nitrotyrosine can serve as a specific marker of peroxynitrite activity and the development of oxidative-nitrosative stress (Bartesaghi and Radi, 2018). We have shown that prednisolone elevated protein nitration at tyrosine residues in the brain tissue by 28% (Tukey’s test, p = 0.0052) compared with the control (Figures 8A, C). This reflects NO-mediated protein damage that may lead to neuronal dysfunction. VD_3_ administration decreased the level of nitrated proteins by 61.7% compared with the prednisolone group (Tukey’s test, p = 0.0005) and by 21% compared with the control (Tukey’s test, p = 0.013). This is clearly consistent with previous data on the antioxidant activity of cholecalciferol (Martín Giménez et al., 2021).

Next, we assessed the degree of oxidative modification of proteins based on the content of carbonyl groups (>C=O), which are formed within protein molecules upon ROS-mediated oxidative modifications of aminoacid residues, especially Pro, Arg, Lys, Trh, as well as a result of the interaction of macromolecules with reactive carbonyl products or reducing sugars (Hauck et al., 2019). Immunoblot analysis of carbonylated proteins did not reveal, in contrast to the level of nitrated proteins, a statistically significant difference between the three groups, as shown in Figures 8B, D. In our opinion, this can be explained by the augmented neutralization of nitric monooxide by superoxide anion radicals with the formation of peroxynitrite, which, in turn, can cause an increase in the content of nitrated proteins in the brain under the GC influence (Figure 8C).

Intensification of ROS and peroxynitrite formation, in addition to protein oxidative modification, can also lead to DNA damage, inducing the development of genotoxic stress. In turn, the appearance of single- and double-strand breaks in DNA activates one of the key enzymes of the cellular DNA repair system, PARP-1, which provides covalent modification of histone and non-histone proteins in the process of their poly(ADP)-ribosylation (Wang et al., 2019). As PARP-1 overactivation was previously reported to be detrimental in multiple types of experimental brain injury, including the impact of oxidative stress (Salech et al., 2020), the brain levels of protein poly(ADP)-ribosylation were studied. We found a significant increase (by 45%, Figures 8E, G) in the level of PAR after prednisolone administration (Tukey’s test, p = 0.00055). As shown in Figures 8F, H, the PARP-1 protein level did not change compared to the control (Tukey’s test, *p* = 0.537) that, however, do not exclude an increase in the overall activity of this enzyme. Interestingly, when comparing rats supplemented with prednisolone and prednisolone plus VD_3_, there were no intergroup differences in PAR (Tukey’s test, *p* = 0.44), suggesting that VD_3_ does not provide effective inhibition of this protein modification. However, VD_3_ supplementation diminished PARP-1 content by 25.3% (Tukey’s test, *p* = 0.015) compared with the prednisolone group, thus affecting protein levels rather than enzyme activity.

Collectively our data suggest that prednisolone caused the development of oxidative-nitrosative stress in brain tissue that may contribute to glucocorticoid-induced neurotoxicity. The ability of VD_3_ to prevent protein nitration and to reduce PARP-1 level was established.

### 3.7. Effect of prednisolone and vitamin D_3_ on glucocorticoid receptor level and NF-κB/IκB pathway in brain tissue

**FIGURE 9:**
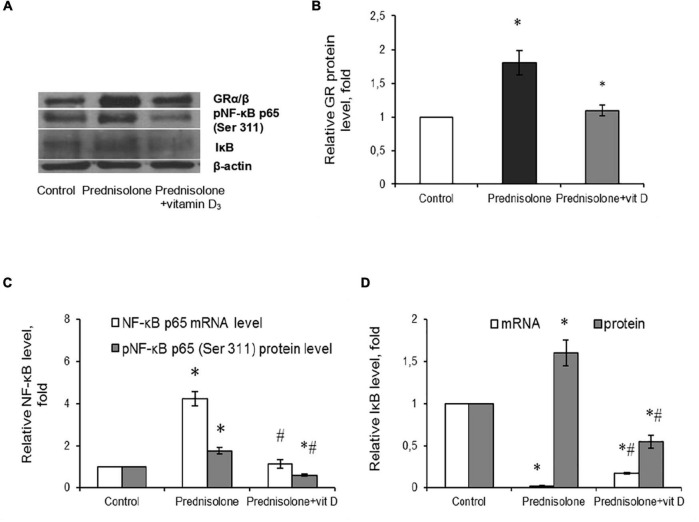
Glucocorticoid receptor level and NF-κB/IκB pathway in brain tissue after prednisolone and vitamin D_3_ administration. Glucocorticoid receptor protein **(A,B)** level was determined by western blot analysis. NF-κB signaling was characterized based on the *Nf*-κ*b p65* mRNA level **(C)**, NF-κB p65 phosphorylated at Ser 311 (Western blot) **(A,C)** and IκB protein **(A,D)** and mRNA **(D)** levels in rat brain tissue of three animal groups: 1—control; 2—prednisolone administration; 3—prednisolone and vitamin D_3_ administration (*n* = 8 rats/group). Protein level was normalized to β-actin and mRNA levels—to *Gapdh* expression. All data are presented as mean ± SD of three independent experiments done in triplicate; *p < 0.05 denotes significance compared with control, ^#^*p* < 0.05 denotes significance compared with prednisolone administration (one-way ANOVA, Tukey’s *post-hoc* test).

Glucocorticoids act through their particular receptor, and in rodents its expression is ubiquitous throughout the brain (Kadmiel and Cidlowski, 2013). It was important to determine whether GR is involved in prednisolone-induced neuropathological changes. Unexpectedly, we observed a significant increase (by 80%, Figures 9A, B) in the GR level after prednisolone administration compared with the control group (Tukey’s test, *p* = 0.01). This may either reflect or contribute to impaired GR-signaling and partially explain the neurotoxic effects of long-term prednisolone administration.

Since NF-κB is reported to be a redox-sensitive transcription factor (Morgan and Liu, 2011), and in addition, GR may interact with NF-κB p65 subunit through direct protein-protein interactions (Ray and Prefontaine, 1994; Nelson et al., 2003), thus influencing its transcriptional activity, we assessed the components of the NF-κB system. Using RT-PCR (Figure 9C), we established a significant 4.23-fold increase in *Nf*-κ*b* p65 mRNA compared with the control rats (Tukey’s test, *p* = 0.0009). The same elevating effect was observed for the NF-κB p65 phosphorylated at Ser311 protein level, which is considered to be transcriptionally competent (Figures 9A, C). The level of phosphoNF-κB p65 was increased by 78.3% after prednisolone compared with the control group (Tukey’s test, *p* = 0.008).

An important role in the regulation of NF-κB p65 activity belongs to the NF-κB inhibitor, which binds to NF-κB dimers and keep them inactive in the cytoplasm (Solt and May, 2008). We studied IκB expression in rat brain and found conflicting results concerning effects of GC on *I*κ*B* mRNA (Figure 9D) and IκB protein (Figures 9A, D) levels. With a strong decrease in the gene expression of IκB under the GC action by 88.2 ± 5.5% (Tukey’s test, *p* = 0.001), the level of IκB protein was 1.6-fold elevated compared with the control (Tukey’s test, *p* = 0.015). This finding suggests GC-induced dysregulation of transcriptional and translational processes in nerve cells. Vitamin D_3_ treatment restored the GR to control values (Figures 9A, B). In addition, VD_3_ almost completely normalized *Nf*-κ*B* mRNA expression and significantly reduced the protein content of phosphoNF-κB p65 (by 66.3% compared vs. prednisolone, and by 40% compared vs. control), suggesting VD_3_ ability to counteract prednisolone-induced NF-κB activation in brain tissue. At the same time, we also observed contradictory effect of VD_3_ supplementation on IκB. It increased *I*κ*B* mRNA (by 9.4-fold compared vs. prednisolone), while decreasing IκB protein level (by 65.6% compared vs. prednisolone, and by 45% compared vs. control). This uncoordinated action of vitamin D_3_ on IκB expression at transcriptional and translational levels renders the entire pattern of NF-κB activation unclear, indicating the need for further study.

To conclude, at the molecular level prednisolone caused an increase in the expression of the p65 subunit of NF-κB and an increase in its specific phosphorylation at Ser311 against the background of an increase in the IκB protein content. VD_3_ diminished the intensity of oxidative-nitrosative stress, possibly by suppressing the activation of NF-κB signaling in the brain.

## 4. Discussion

A preventive approach to diminish the side effects of synthetic GCs on CNS may be achieved by using GC doses as low as possible, and by modulating the duration of the therapy, especially in high-dose courses. However, evidence has emerged, that synthetic GC neuropsychiatric sequelae often requiring psychiatric treatments that are not presently standardized. Moreover, little attention has been paid to developing possible supportive therapeutic strategies that can be applied along with the GC course. Therefore, the main scientific goal of the study was to solve the urgent medical and biological problem how to prevent and/or manage the neurotoxicity caused by long-term GC administration, in particular prednisolone, which is still widely used in clinical practice for the treatment of chronic inflammatory diseases.

Therapeutic administration of synthetic glucocorticoids may lead to adverse psychiatric effects (steroid-induced psychosis), including depressed mood, emotional lability, and even suicidality. Despite of synthetic GC therapy is frequently associated with a variety of clinical manifestations on behavior, mood and cognition, the real incidence is not presently inferable from the available literature, because few validated studies have been performed (Fietta et al., 2009). Therefore, we focused on the assessment of possible prednisolone-associated behavioral changes. Despite the fact that prednisolone is one of the most widely used synthetic glucocorticoids for the treatment of various allergic, inflammatory and autoimmune diseases, to the best of our knowledge, there have been no animal experiments focused on both prednisolone-induced behavioral changes in the brain and uncovering their functional, cellular and molecular basis.

Generally, GCs affect mood, and cognition (Keenan et al., 1996) and cause sleep disturbances (Curtis et al., 2006) in a dose- and time-dependent manner. In rodents, repeated administration of corticosterone induced depression-like behaviors (Kalynchuk et al., 2004; Zhao et al., 2008). Regarding prednisolone, there was one study on mice reporting that prednisolone (50–100 mg/kg, 7 days) caused anxiety- and depression-like behavioral alterations and impaired expression of apoptotic genes in hippocampus (Kajiyama et al., 2010). In contrast, in treatment-resistant depressed patients with fatigue and hypocortisolaemia, an augmentation of antidepressant therapy with synthetic GC prednisone may be useful and patients demonstrated significant improvement in depression (Bouwer et al., 2000).

Results from our set of behavioral experiments indicted that prednisolone did not affect locomotor activity (based on OFT) or learning and memory (based on contextual/cued conditioning). In contrast, at least one study demonstrated that a low daily dose of methylprednisolone (5 mg/kg) administered for 10-days favors aversive memory persistence in adult rats, without affecting the exploring behavior, locomotor activity, anxiety levels, and pain perception (de Vargas et al., 2017). Herein, we found that prednisolone caused depressive-like changes in behavior (based on FST) along with possibly anti-anxiety behavior (based on EPM), if considering that the more time spent in the open arms corresponds to the lower level of anxiety. These data are in line with the finding that in patients with chronic GC treatment depressive disorder is the most frequent diagnosis, while anxiety disorder, mania or psychosis are less common (Lin et al., 2020). Moreover, depression-like behavior was induced by subcutaneous treatment of prednisolone in male mice (Aoki et al., 2022). Interestingly, there is a relationship between EPM and FST. Studies performed with selectively bred animals, found association between low exploration of the plus-maze open arms and high immobility in the FST (Keck et al., 2001; Estanislau et al., 2011). However, we found the reverse correlation between FST and EPM after prednisolone treatment: with a high immobility time in FST there was a high exploration of the plus-maze open arms in EPM. Therefore, based on possible discrepancies in the interpretation of EPM data, we are inclined to believe that changes in EPM parameters may more likely reflect a panic reaction, especially in combination with an increase in the time of immobility in the FST, than a lower level of anxiety.

As reported previously, chronic exposure to moderate/high doses of synthetic GCs may induce cumulative and potentially long-lasting effects on specific brain area morphology, such as hippocampus (Brown et al., 2004) and amygdala (Brown et al., 2008) volumes. In a study at the histological level we addressed the question of which brain areas and to what extent are affected by prednisolone. Generally, the cytoarchitectonics of the cerebral cortex and the posterior nucleus of the thalamus after prednisolone load were preserved and no valuable differences compared with the control animals were found in our trial. Previously, the influence on prefrontal cortex was found for naturally occurring glucocorticoids, however, to our knowledge there is a lack of data related to synthetic GCs. The only study we found postulated that the corticosteroid prednisolone increased amygdala and insula reactivity to food approach signals (Serfling et al., 2019). Regarding the thalamus, authors of this study (Yamazaki et al., 2021) suggested that GC administration reduced cerebral blood flow in the hippocampus and thalamus in dogs, similar to that which occurs in humans.

In contrast to cortex and thalamus, the effects of GCs on the hippocampus are well described (Höschl and Hajek, 2001). Our data confirmed previous studies revealing that prednisolone caused a slight reduction in cell density in the hippocampal areas. We additionally showed an increase in the area of the perikaryon and the size of the nuclei in CA1–CA3 areas. Such a pronounced effect of GCs on hippocampus may be explained by the high levels of GR expression in the paraventricular nucleus of the hypothalamus and in the hippocampus (Wang et al., 2013), both in glial cells and neurons, suggesting the high sensitivity of these brain structures to the GC exposure. In contrast to our expectations, we also observed a significant elevation of GR protein level in the brain, possibly, in the hippocampus, after prednisolone treatment that may contribute to GC-induced brain lesion.

Previously, it was shown that GC therapy may disrupt cerebellar development through the rapid induction of apoptosis in the cerebellar external granule layer (Noguchi, 2014). Since, clinical research also suggests the cerebellum may be particularly susceptible to GC exposure, we also performed the histological investigation of cerebellum, which is primarily responsible for the locomotor function, coordination, balance and posture control, muscle tone, and motor learning. Surprisingly, despite the fact that prednisolone did not affect locomotor activity, we found a significant decrease in the size of neurons in the cerebellar cortex. Since neuronal size is correlated with the extent of a neuron’s dendritic and axonal arbor, a decrease in somal size may reflect decreased afferent and/or efferent connectivity of these cells that altogether may indicate the onset of neurodegenerative changes in the cerebellum under the influence of prednisolone.

The next studied level was to characterize functional abnormalities based on assessment of synaptic characteristics: the amplitude of exocytosis stimulated by high K^+^, the rate of Ca^2+^-dependent fusion of isolated synaptic vesicles with synaptic plasma membranes of nerve terminals and the level of LTP in CA1–CA3 hippocampal synapses. We established a GC-induced decrease in all three parameters, reflecting the impaired processes of depolarization-induced exocytosis in rat synaptosomes, altered membrane fusion and LTP. The decrease in the amplitude of high-K^+^ stimulated exocytosis reflects the changes in the synaptic vesicle fusion competence and/or their readiness for release. This, in turn, affects the concentration of the neurotransmitters and their receptor activation at the post-synaptic membrane. As previously shown in a rat model of vitamin D_3_ deficiency, synaptic vesicle acidification did not change and no alterations were observed at the endocytotic stage (Kasatkina et al., 2020). One of the reasons for a decreased amplitude of exocytosis, established in this study, was linked to changes in the rate of synaptic vesicle fusion with plasma membranes, which, in turn, depended on the cholesterol level in both synaptic plasma membranes and synaptic vesicles. Impaired cholesterol turnover in the brain can be one of the possible contributors to GC-associated brain disorder, since cholesterol level was markedly elevated in synaptic plasma membranes isolated from cerebral hemispheres after prednisolone action. These data are consistent with the previously shown ability of prednisone, similar to prednisolone, to cause an increased serum cholesterol even after 2 days of the treatment (Taskinen et al., 1988). Another evidence of the involvement of cholesterol impairments in the pathogenesis of GC-induced brain lesion is that defects in brain cholesterol metabolism may be implicated in the pathology of neurodegenerative diseases, such as Alzheimer’s disease, Parkinson’s disease, and some cognitive deficits typical of the old age. Neurons were also found to increase cholesterol synthesis in chronic myelin disease and multiple sclerosis (Berghoff et al., 2021).

The hippocampus is one of the brain regions mostly affected by stressful situations and GC treatment. A variety of physiological synaptic plasticity phenomena, which can be electrophysiologically perceived as long-term potentiation is involved in memory formation in the hippocampus (Lynch, 2004). At the same time, along with histological alterations in hippocampus, prednisolone caused a reduced fEPSP in CA1–CA3 hippocampal synapses, reflecting the interference with hippocampal electrophysiological activity, which in turn, may underlie the observed behavioral changes. Thus, our data confirmed that glucocorticoids are known to suspend the induction of LTP (Maggio and Segal, 2007). However, it was shown predominantly for naturally occurring GCs (Krugers et al., 2005). In contrast to prednisolone action on LTP, the following study demonstrated an enhanced LTP, a phenomenon that was strengthen in hippocampal slices of rats injected with synthetic GC methylprednisolone (5 mg/kg) during 10 days (de Vargas et al., 2017), emphasizing that the influence of GC on LTP is tightly mediated by treatment doses and duration.

One of an important issue of our study was to discover the possible molecular mechanisms underlying glucocorticoid-induced brain lesion at both histological and functional levels. Growing evidence suggest that reactive oxygen and nitrogen species are one of the major contributors to the neurotoxicity induced by exogenous toxins (Singh et al., 2019). Moreover, ROS/RNS (singlet oxygen, superoxide and peroxynitrite) may modulate the effects of VD_3_ by inhibiting association of 1,25(OH)_2_D_3_-VDR with DNA, inducing irreversible VDR inhibition (Kröncke et al., 2002). Indeed, in the case of prednisolone load, we observed the development of oxidative-nitrosative stress in brain tissue that may contribute to glucocorticoid-induced brain damage. Oxidative-nitrosative stress was observed with a simultaneous increase in the expression and phosphorylation of the p65 NF-κB subunit, in contrast to the large pool of evidences on the inhibitory effects of GCs on NF-κB (De Bosscher et al., 2000; Nelson et al., 2003).

Since we assumed that one of the contributors that enhances the damaging effect of GCs on the brain is an abnormal VD_3_ status, we examined the effect of prednisolone on the state of the VD_3_-auto/paracrine system. It has been shown for the first time that prednisolone causes a profound depletion of the circulating 25OHD_3_ pool that may be associated with a decrease in VDBP, the main transport protein and vitamin D_3_ depot. Previously, we showed that prednisolone may cause a decrease in 25OHD_3_, but only in serum (Lisakovska et al., 2020). A long-term vitamin D_3_ deficiency in GC-administered animals strongly affected the functioning of the brain VD_3_-auto/paracrine system. Elevated VDR expression, as well as CYP27B1, which converts 25OHD_3_ to the hormonally active 1,25(OH)_2_D_3_, in the brain may represent, most likely, a compensatory tissue response to a decrease in the main circulating vitamin D metabolite, 25OHD_3_, in serum, and most importantly—in cerebrospinal fluid and brain tissue. Finally, an adaptive response to impaired transport and reduced levels of 25OHD_3_ and 1,25(OH)_2_D_3_ in the brain also manifested in a significant decrease in catabolic enzyme CYP24A1 that might preserve a pool of hormonally active form of VD_3_. Thus, the novelty of this study lies in the fact that for the first time the circulating pool of vitamin D_3_ and the state of the VD_3_-auto/paracrine system in the brain are characterized more thoroughly on the rodent model of chronic prednisolone administration.

Given the accumulating evidence that optimal vitamin D_3_ levels are required to maintain healthy adult brain function, the logical decision was to correct the VD_3_ deficiency status with vitamin D_3_ supplementation to reduce brain-related manifestations of GC-induced neurotoxocity. As expected, after taking cholecalciferol, we observed a complete normalization of the 25OHD_3_ content in the serum and a partial – in the CSF and brain tissue. Our results suggest that complete repletion of the 25OHD_3_ pool in the brain, likely, takes time and requires longer treatment with higher doses of VD_3_. In addition, it should be noted that serum levels of 25OHD_3_ are typically stabilized within three months after VD_3_ deficiency treatment (Gani and How, 2015). Thus, we can speculate that the 25OHD_3_ level we observed in the brain and cerebrospinal fluid may still be low and insufficient for the local needs of the brain after 30 days of cholecalciferol treatment.

Among all components of the VD_3_-auto/paracrine system, only the VDBP returned to the control value. This may be caused by its important role not only as the main transport protein for VD_3_ metabolites, but also by its involvement in extracellular actin sequestration and activation of the complement system that makes VDBP responsible for neuroimmune homeostasis (Gomme and Bertolini, 2004). However, difficulties in explaining the changes in VDBP pattern after GC and cholecalciferol supplementation may be due to the fact that it is still unclear whether VDBP is synthesized directly in the CNS or transported from the general circulation through the BBB (Kim et al., 2021).

It is quite logical that after partial repletion of 25OHD_3_ we observed a decrease in the level of VDR mRNA and protein compared with both prednisolone and control animals that obviously, should have provided adequate calcitriol signaling. Previously, we showed the same effect of reducing VDR after the VD_3_ administration against the background of prednisolone load in various tissues and organs, including bone marrow (Shymanskyi et al., 2018) and liver (Lisakovska et al., 2017), as well as kidneys (Mazanova et al., 2022) and liver (Mazanova et al., 2018) in diabetes-associated VD_3_ deficiency. In addition, we revealed a marked decrease in CYP27B1 mRNA compared with prednisolone animals, most likely, in response to partially depleted pool of 25OHD_3_, thereby maintaining balance in the synthesis of the hormonally active form of VD_3_. Surprisingly, the 24-hydroxylase content remained unchanged. Thus, an adequate response of the VD_3_-auto/paracrine system after supplementation with VD_3_ may provide adequate 25OHD_3_/1,25(OH)_2_D_3_-mediated functions in the brain.

The presence of VDR in the hippocampus, cortex and limbic systems of humans and rodents support an important role of VD_3_ in regulating learning, memory, mood and cognitive performance (Gáll and Székely, 2021). According to our results, vitamin D_3_ treatment caused anti-depressive effect and reliably restored exploratory behavior in the EPM after the development GC-induced neurotoxicity, suggesting that GC-associated behavioral changes may be reversible after restoration of VD_3_ status. In support to our data, beneficial effects of vitamin D_3_ on anxiety and depression-like behavior induced by unpredictable chronic mild stress by suppression of brain oxidative stress and neuroinflammation in rats was shown by Bakhtiari-Dovvombaygi et al., 2021. Moreover, it was reported that VD_3_ supplementation along with increased dairy-product intake exerted a significant positive impact on physical and mental health status in psychiatric patients (Abdul-Razzak et al., 2018). However, previous studies regarding the use of VD_3_ and its role in prevention and treatment of depressive disorders included small number of people to clearly assess the effectiveness and safety of vitamin D_3_ as adjunctive therapy to antidepressants, as well as and its dosage range (Stefanowski et al., 2017). Therefore, our study may be considered one more evidence to support the use of vitamin D_3_ as an antidepressant adjunctive therapy in different pathologies associated with VD_3_ deficiency and depressive state.

In contrast to our data of no changes in OFT after VD_3_ treatment, it was reported that vitamin D_3_ deficiency in adult male mice results in hyperlocomotion in a novel OFT (Groves et al., 2013). However, these discrepancies can be explained by the different causes of VD_3_ deficiency – induced by GC in our study and caused by vitamin D_3_-deficient diet. Considering contextual and cued conditioning, there were also no effects after cholecalciferol treatment, consistent with the finding that VD_3_ status did not affect these parameters. In particular, Harms et al., 2012 showed that VD_3_ deficiency does not affect the acquisition or retention of cued fear conditioning, nor does it affect the expression of latent inhibition using a fear conditioning paradigm.

At the histological level, vitamin D_3_ exerted its effects predominantly on the hippocampus, influencing the size of the perikaryon and nucleus in the CA1 area, and to a lesser extent in the CA2/CA3 regions. This can be explained by a large number of VDR-expressing cells in the hippocampus (Gezen-Ak et al., 2013). Interestingly, despite the presence of VDR in the thalamus and cerebellum, no valuable effects of VD_3_ on the cytoarchitectonics of the thalamus and cerebellum were found.

Not surprisingly, the most pronounced normalizing effect of VD_3_ was observed at the functional level, since 1,25(OH)_2_D_3_ is classified as a neurosteroid, which can alter neuronal excitability (Eyles, 2020). We found recovery of depolarization-induced synaptic vesicle fusion along with normalization of cholesterol levels after VD_3_ treatment, suggesting reversibility of GC-induced changes in stimulated secretion. Vitamin D_3_ has previously been shown to maintain a balanced excitatory and inhibitory neurotransmission in an animal model of VD_3_-deficiency (Kasatkina et al., 2020). Furthermore, in our study, vitamin D_3_ treatment restored prednisolone-induced LTP reduction, suggesting the ability of cholecalciferol to influence synaptic plasticity, consistent with the finding that optimal vitamin D_3_ levels are required for LTP induction (Salami et al., 2012). Thus, our data highlight the critical role of VD_3_ in restoring basic synaptic transmission and synaptic plasticity in GC-evoked brain lesion.

Vitamin D_3_/VDR is known to play a protective role in diseases associated with oxidative stress and inflammation by acting on various cellular pathways through the regulation of gene expression and epigenetic modifications. One possible mechanism for the neurotropic action of VD_3_ has been reported to involve ROS/RNA suppression (Sepidarkish et al., 2019). We also confirmed that against the background of chronic GC intake, vitamin D_3_ showed antioxidant effects, manifested in a reduced intensity of oxidative-nitrosative stress. Our data are consistent with a study showing that calcitriol can reduce NO levels by inhibiting the expression of inducible NO synthase in the spinal cord and brain (Garcion et al., 2002). Moreover, 1,25(OH)_2_D_3_ significantly affects the synthesis of cytokines in various tissues, which at the cellular level realize their action through the VDR-mediated regulation of NF-κB activity. It has been shown that VDR can physically interact with IκB-β kinase to block NF-κB activation (Chen et al., 2013). However, there is currently insufficient available research investigating the effects of VD_3_ on various aspects of NF-κB functioning in the brain, especially those related to the mechanisms of GC-related neurotoxicity. Overall, in the present study, we found the ability of vitamin D_3_ to suppress GC-induced activation of NF-κB signaling in the brain, which may lead to an improvement in GC-induced deleterious brain changes, thereby conferring the neuroprotective effects.

The study has the following limitations. All protein levels were measured on whole brain lysates that complicates further analysis. It was previously found that the baseline levels of the components of the VD_3_-auto/paracrine system differ depending on the brain area, for example, the content of VDR and CYP24A1 mRNA is higher in hippocampal neurons than in the cortex (Gezen-Ak et al., 2013). Therefore, it can be assumed that the observed changes after taking prednisolone and VD_3_ may reflect a multidirectional response from different cell types and brain regions. To solve this problem, in the following experiments we will study changes in different areas of the brain, dissecting separately the cortex, hippocampus, cerebellum and thalamus. This issue also applies to the need for brain cell typing, since the potential protective role of NF-κB in neurons must be differentiated from its potential degenerative role in glia, but this assumption definitely needs further study.

Despite the stated shortcomings, the strength of our research study lies in the comprehensive study that characterizes CNS damage after long-term exposure to the widely used GC prednisolone, manifested at the molecular, functional, histological and behavioral levels in the same animal model. The study provides insight into the possible mechanisms of glucocorticoid-associated neurotoxicity and neuroprotective action of vitamin D_3_ that is important for future clinical applications. The results of this fundamental research will serve as a theoretical basis for evidence-based practical recommendations on the use of vitamin D_3_ in the treatment of brain damage and cognitive dysfunctions associated with long-term GC supplementation, as well as in the treatment of other nervous and metabolic diseases accompanied by impaired VD_3_ metabolism. In our opinion, the further perspective of this study should be devoted to the search for the optimal treatment duration and cholecalciferol dosage that will more fully restore the circulating pool of 25OHD_3_ in the CNS without affecting calcium metabolism and bone homeostasis.

## 5. Conclusion

Our findings indicate that prednisolone-induced neurotoxicity and behavioral disturbances in rats are associated with the VD_3_ deficiency and alterations in the VD_3_-auto/paracrine system. Replenishment of the circulating 25OHD_3_ pool and partial normalization of the VD_3_-auto/paracrine system after vitamin D_3_ supplementation had a neuroprotective effect. Vitamin D_3_ decreased oxidative-nitrosative stress and NF-κB activation, normalized the morphometric parameters of hippocampal neurons, restored depolarization-induced fusion of synaptic vesicles with synaptic plasma membrane, as well as LTP in the hippocampus that led to an antidepressant-like effect of the treatment. Thus, vitamin D_3_ may be effective in the prevention of molecular, structural, functional abnormalities and behavioral disorders caused by chronic administration of prednisolone.

## Data availability statement

The original contributions presented in this study are included in the article/supplementary material, further inquiries can be directed to the corresponding author.

## Ethics statement

This animal study was reviewed and approved by the Ethics Committee on controlling the rules of research work with experimental animals, Palladin Institute of Biochemistry, National Academy of Sciences of Ukraine, Kyiv, Ukraine.

## Author contributions

OL and IS conceived the original idea, designed and supervised the study, interpreted data, and prepared the manuscript. IS coordinated the research group. OL, IS, DL, AK, LK, AS, and SS performed the laboratory experiments. DI, DL, and OL performed the behavioral tests. OL, DL, AS, and DI statistically analyzed the data. DI and LK contributed to the critical discussion and data analysis. MV made a substantial contribution to the general conception of the work and critically revised the manuscript. All authors read and approved the final manuscript.
